# Wearable Devices for Supporting Chronic Disease Self-Management: Scoping Review

**DOI:** 10.2196/55925

**Published:** 2024-12-09

**Authors:** Marie-Pierre Gagnon, Steven Ouellet, Eugène Attisso, Wilfried Supper, Samira Amil, Caroline Rhéaume, Jean-Sébastien Paquette, Christian Chabot, Marie-Claude Laferrière, Maxime Sasseville

**Affiliations:** 1 Faculty of Nursing Sciences Université Laval Québec, QC Canada; 2 VITAM Research Center on Sustainable Health Québec, QC Canada; 3 School of Nutrition Université Laval Québec, QC Canada; 4 Department of Family Medicine and Emergency Medicine Faculty of Medicine Université Laval Québec, QC Canada; 5 Research Center of Quebec Heart and Lungs Institute Québec, QC Canada; 6 Patient Partner VITAM Research Center on Sustainable Health Québec, QC Canada; 7 Library Université Laval Québec, QC Canada

**Keywords:** chronic diseases, self-care, self-management, empowerment, mobile health, mHealth, wearable, devices, scoping, review, mobile phone, PRISMA

## Abstract

**Background:**

People with chronic diseases can benefit from wearable devices in managing their health and encouraging healthy lifestyle habits. Wearables such as activity trackers or blood glucose monitoring devices can lead to positive health impacts, including improved physical activity adherence or better management of type 2 diabetes. Few literature reviews have focused on the intersection of various chronic diseases, the wearable devices used, and the outcomes evaluated in intervention studies, particularly in the context of primary health care.

**Objective:**

This study aims to identify and describe (1) the chronic diseases represented in intervention studies, (2) the types or combinations of wearables used, and (3) the health or health care outcomes assessed and measured.

**Methods:**

We conducted a scoping review following the Joanna Briggs Institute guidelines, searching the MEDLINE and Web of Science databases for studies published between 2012 and 2022. Pairs of reviewers independently screened titles and abstracts, applied the selection criteria, and performed full-text screening. We included interventions using wearables that automatically collected and transmitted data to adult populations with at least one chronic disease. We excluded studies with participants with only a predisposition to develop a chronic disease, hospitalized patients, patients with acute diseases, patients with active cancer, and cancer survivors. We included randomized controlled trials and cohort, pretest-posttest, observational, mixed methods, and qualitative studies.

**Results:**

After the removal of 1987 duplicates, we screened 4540 titles and abstracts. Of the remaining 304 articles after exclusions, we excluded 215 (70.7%) full texts and included 89 (29.3%). Of these 89 texts, 10 (11%) were related to the same interventions as those in the included studies, resulting in 79 studies being included. We structured the results according to chronic disease clusters: (1) diabetes, (2) heart failure, (3) other cardiovascular conditions, (4) hypertension, (5) multimorbidity and other combinations of chronic conditions, (6) chronic obstructive pulmonary disease, (7) chronic pain, (8) musculoskeletal conditions, and (9) asthma. Diabetes was the most frequent health condition (18/79, 23% of the studies), and wearable activity trackers were the most used (42/79, 53% of the studies). In the 79 included studies, 74 clinical, 73 behavioral, 36 patient technology experience, 28 health care system, and 25 holistic or biopsychosocial outcomes were reported.

**Conclusions:**

This scoping review provides an overview of the wearable devices used in chronic disease self-management intervention studies, revealing disparities in both the range of chronic diseases studied and the variety of wearable devices used. These findings offer researchers valuable insights to further explore health care outcomes, validate the impact of concomitant device use, and expand their use to other chronic diseases.

**Trial Registration:**

Open Science Framework Registries (OSF) s4wfm; https://osf.io/s4wfm

## Introduction

### Background

Noncommunicable diseases, commonly referred to as chronic diseases, are the leading cause of death worldwide, responsible for 41 million deaths annually and accounting for 74% of global mortality [1]. Aging populations, combined with unhealthy lifestyles such as poor diet and sedentary habits, contribute to a significant rise in chronic disease risk factors, including hypertension, hyperglycemia, and hyperlipidemia [1]. These diseases are characterized by their long-term nature, slow progression, and need for continuous care and self-management [2]. Most patients with a chronic disease in primary care have at least one additional chronic condition, making multimorbidity (defined as 2 concomitant chronic diseases) a common challenge that requires integrated, patient-centered care approaches to reduce the treatment burden [3].

Self-management is a key element of the care plan, and capturing meaningful information to empower individuals with chronic diseases is essential. Technological advancements have paved the way for wearable electronic devices and sensors that empower individuals to manage their health conditions more independently [4,5]. These innovations support the adherence to disease-related recommendations, such as medication regimens and symptom monitoring, by facilitating real-time monitoring and data collection. Wearable devices operate as data collection tools, transmitting information to software applications for analysis and delivering actionable health updates or notifications [6]. Key features of these devices include (1) automatic data collection (eg, blood glucose [BG], blood pressure [BP], physical activity, and heart rate); (2) direct transmission of data to the patient and, in some cases, their primary health care providers; and (3) availability of data across multiple platforms (eg, smartwatches, smartphones, and monitors). These systems offer real-time feedback either automatically or via clinician intervention, promoting sustained patient engagement with recommended health behaviors [4-6].

This potential for patient empowerment using wearables is observed in several reviews that report positive effects on clinical outcomes, such as glycemic control, and behavioral outcomes, such as symptom self-management [7-15]. Systematic reviews have explored the efficacy of wearables in managing diabetes, especially in relation to glycemic control [8-15]. BG monitoring devices, a common wearable for diabetes management, exemplify the utility of these technologies in facilitating behavior change and adherence to treatment protocols [8-15]. Numerous literature reviews have explored the use of wearable technologies in chronic disease management focusing on specific devices and health outcomes. Existing literature reviews are focused on specific wearable technologies, such as activity trackers [16-20] or BG monitors [8,9,11], or on self-management within particular chronic disease populations, including patients with diabetes [8-15,21], cardiovascular diseases [22,23], and cancer [7] and cancer survivors [17], as well as populations at risk of chronic diseases [19,24].

Several reviews have emphasized the impact of wearables on specific health outcomes, such as increased physical activity [16,17,19,20]. While these focused reviews offer valuable insights, they often do not capture the broader implications of wearable technology across multiple chronic conditions and diverse health outcomes. Despite the growing body of research, few comprehensive reviews have examined the intersection of various chronic diseases, wearable devices, and the range of health outcomes assessed in intervention studies. A systematic review identified a gap in understanding how wearables influence health outcomes in chronic diseases despite their potential for enhancing self-management [25]. To address this gap, Mattison et al [25] conducted a comprehensive review across multiple chronic diseases and outcome measures, highlighting the importance of such an analysis particularly in the context of primary health care.

Building on this work, our review adopted broad objectives aiming to provide an overview of the role of wearables in chronic disease management considering the clinical realities of the variety of devices used and the high prevalence of multimorbidity. A comprehensive analysis of wearable devices across various chronic diseases and health outcomes is essential for future research and patient-centered interventions in primary care settings that will reduce the treatment burden.

### Objectives

The objectives of this scoping review were to identify and describe (1) the chronic diseases represented in intervention studies, (2) the types or combination of wearables used, and (3) the health or health care outcomes assessed and measured. This scoping review uniquely presents a comprehensive overview of the populations of patients with chronic diseases involved, the wearable devices used, and the specific outcomes targeted in intervention studies.

## Methods

### Overview

We conducted a scoping review informed by the Joanna Briggs Institute guidelines [26] and reported our results based on the PRISMA-ScR (Preferred Reporting Items for Systematic Reviews and Meta-Analyses extension for Scoping Reviews) checklist [27]. We registered the review protocol in the Open Science Framework Registries [28]. We followed the population, intervention, comparator, and outcome framework [29] in establishing the study selection criteria (Textbox 1).

**Population, intervention, comparator, outcome, time frame, and study design framework elements [29].**

**Inclusion criteria**

- Population: adult populations (aged ≥18 years) with chronic diseases (diseases characterized by their long-term nature, slow progression, and need for continuous care [2]).
- Intervention: interventions using wearable devices or smart health devices, such as connected weighing scales, that automatically collect and transmit biological or behavioral data to patients
- Comparator: usual care (without a wearable device)
- Outcomes: clinical, behavioral, patient experience with technology, holistic or biopsychosocial, and health care system outcomes
- Time frame and study design: between 2012 and 2022; randomized controlled trials, quasi-randomized controlled trials, prospective cohort studies, pretest-posttest studies, observational studies, mixed methods studies, and qualitative studies; published in English or French

**Exclusion criteria**

- Population: participants aged <18 years; patients with acute diseases; hospitalized patients; patients with active cancer and cancer survivors; participants with only a predisposition to develop a chronic disease, such as obesity, prediabetes, and prehypertension (without a diagnosis of dyslipidemia or another chronic disease)
- Intervention: hospital inpatient setting; no automatic data collection or transmission by the device, manual device, or survey instrument; data transmission only to caregivers or researchers (not to patients); studies only involving glucose monitoring devices and only measuring glucose levels as an outcome; automated insulin delivery system, also known as “closed loop” control system
- Comparator: not applicable
- Outcomes: Outcomes not related to chronic diseases; studies only addressing development or validation outcomes
- Time frame and study design: reviews, surveys, quantitative descriptive studies, metrology or diagnostic studies, protocols or ongoing studies, and conference papers

We included interventions involving at least one wearable device that automatically collected and transmitted data to adult populations with at least one chronic disease in a primary health care setting. The term “wearables” refers to all devices that collect patient data by directly capturing physiological signals, including connected weighing scales, electronic medication pillboxes, and ingestible sensors. Chronic diseases are defined as health conditions that are likely permanent and can be managed in primary health care settings. We excluded studies involving participants who only had a predisposition to develop chronic diseases, hospitalized patients, individuals with acute diseases, patients with active cancer, and cancer survivors.

Our review included randomized controlled trials (RCTs), cohort studies, pretest-posttest studies, observational studies, mixed methods studies, and qualitative studies published between 2012 and 2022. We excluded reviews, surveys, quantitative descriptive studies, metrology or diagnostic studies, protocols or ongoing studies, and conference papers. Moreover, we modified our published protocol [28] to exclude studies focused solely on the efficacy of connected glucometers that only measure glucose levels as there is already a strong evidence base on this topic [8-15].

### Search Strategy

We (MPG and MS) developed search strategies in the MEDLINE (Ovid) and Web of Science (Science Citation Index Expanded, Social Sciences Citation Index, Arts and Humanities Citation Index, and Emerging Sources Citation Index) databases with an experienced librarian (MCL) to identify sources published between January 1, 2012, and June 30, 2022. The search strategies can be found in Multimedia Appendix 1.

### Data Collection

We imported all references on June 30, 2022, to the web-based collaborative tool Covidence (Veritas Health Innovation) [30] and removed duplicates manually and using the automated function in Covidence before screening. We conducted a calibration exercise with the reviewers (Victoria Bureau Lagarde, EA, WS, SO, Rouwayda Elouni, and SA) and 2 senior researchers (MPG and MS) using a sample of 10 sources to ensure consistency in the application of the inclusion and exclusion criteria before the full screening process. At the first *title and abstract* screening phase, 8 reviewers (Victoria Bureau Lagarde, EA, WS, SO, MPG, MS, Rouwayda Elouni, and SA) independently assessed the titles and abstracts of the sources by applying the inclusion and exclusion criteria. All titles and abstracts underwent dual screening. Discrepancies in decisions were resolved through team consensus. At the *full-text review* stage, we searched and obtained all missing full texts of the selected references and imported them into Covidence. We conducted another calibration exercise with 10 full texts; 7 reviewers (SO, WS, MS, MPG, Victoria Bureau Lagarde, SA, and EA) independently applied the same selection criteria, and all exclusion motives were recorded in Covidence. All full texts underwent dual screening, and any discrepancies were also resolved through team consensus. From the included texts to extract, we tagged those related to the same studies and combined them for extraction. We also performed backward hand searching [31] for relevant articles in the bibliographies of the included studies and added them to Covidence for screening and extraction.

### Data Extraction

Following the Joanna Briggs Institute recommendations [32], we developed a grid to extract relevant data from the included studies. We tested this grid in a meeting with reviewers (SO, WS, and EA) and 2 senior researchers (MPG and MS). The data extraction of each included study was performed once by 3 different reviewers (SO, WS, and EA) and validated by at least one senior researcher (MPG or MS). In Covidence, we extracted general information (eg, title of the paper, year of publication, lead author, country, and potential author conflicts of interest), methods (eg, RCT, nonrandomized experimental study, cohort study, pretest-posttest study, mixed methods study, qualitative research, or economic evaluation), intervention data (duration of the intervention and follow-up), setting data (where patients were sourced from), participant data (eg, target population, chronic disease or chronic diseases, inclusion and exclusion criteria, sampling methods, recruitment methods, and methods of allocation to the intervention group), total number of participants included in the analysis sampling (at the end of the study), participant baseline characteristics, process of the intervention (eg, wearable devices, intervention administration, type of blinding, comparator administration, and control group description), all outcomes measured and how they were measured, and intervention effects or results.

### Quality Assessment

We imported the assessment questions from the Mixed Methods Appraisal Tool (MMAT) [33] into Covidence. According to the MMAT, each study is assigned a score that ranges from 0 to 5 stars, with 5 stars signifying the highest quality and reflecting the rigor of the study’s methodology. The quality of each included study was assessed once by 3 different reviewers (SO, WS, and EA), with each study’s score validated by at least one senior researcher (MPG or MS). Discrepancies were resolved through consensus among the reviewers and a senior researcher.

### Data Synthesis

We structured our analysis according to chronic disease clusters; types and combination of wearable devices used; categories of health and health care system outcomes; and reported effects in terms of positive (reported in the studies as statistically significant), neutral, or negative. We synthesized data using tables explained through a narrative approach. We classified the outcomes reported in the intervention studies into 5 categories. The first category involves clinical outcomes, including indicators measured, such as glucose levels (glycated hemoglobin [HbA_1c_]), BP, heart rate, weight, BMI, sleep time and quality, and pain intensity. The second category includes behavioral outcomes, such as physical activity levels, activation, motivation, self-efficacy, and disease-related knowledge. The third category involves patient technology experience, such as satisfaction, use, adherence, and engagement. The fourth category includes holistic and biopsychosocial outcomes, such as health status, health-related quality of life (QoL), physical well-being, anxiety, and depression. The fifth category involves the health care system, such as effectiveness, cost-effectiveness, admission or readmission to hospital, and emergency department visits.

## Results

### Overview

**Figure 1:**
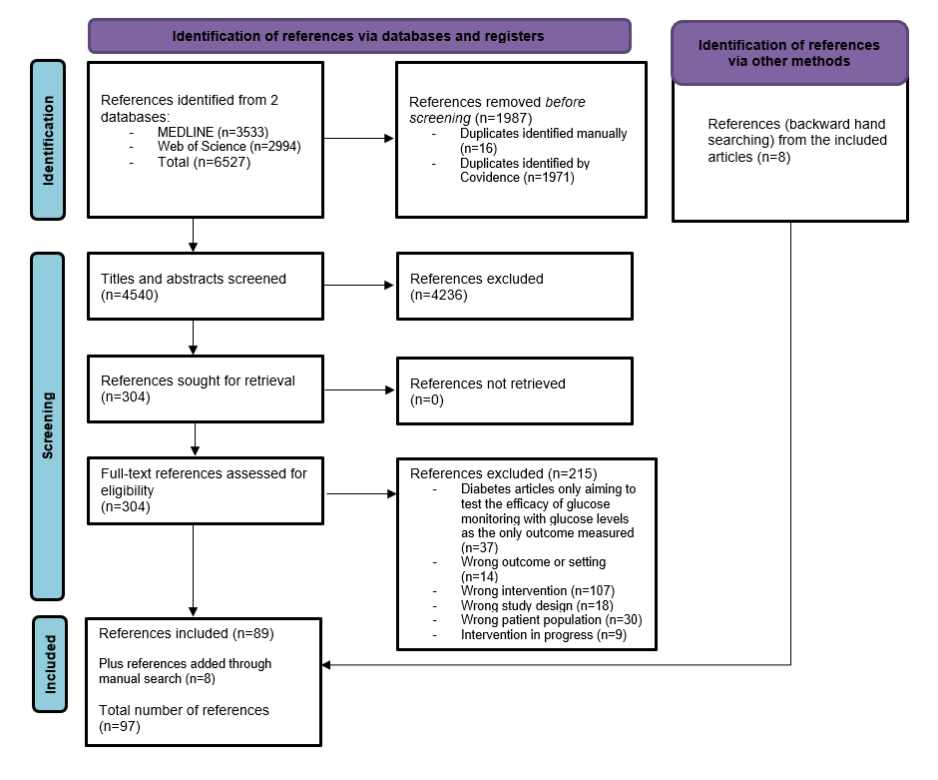
PRISMA (Preferred Reporting Items for Systematic Reviews and Meta-Analyses) flow diagram.

After the removal of 1987 duplicates (n=1971, 99.19% through Covidence and n=16, 0.81% manually), we screened 4540 titles and abstracts. Of the remaining 304 references after exclusion, we excluded 215 (70.7%) full texts and included 89 (29.3%). Of these 89 texts, we tagged 10 (11%) [34-43] related to same intervention studies and combined them for extraction, resulting in 79 included studies. We also hand searched and found 8 relevant articles [44-51] related to the included studies, totaling 97 references: 10 (10%) combined articles [34-43], 8 (8%) hand searched articles [44-51], and 79 (81%) main articles of the included studies [52-130]. The PRISMA (Preferred Reporting Items for Systematic Reviews and Meta-Analyses) 2020 flow diagram [131] of the study inclusion process can be consulted in Figure 1.

### Characteristics of the Included Studies

Of the 79 studies included, some were published in 2 or even 3 scientific articles. As a result, the number of references (n=97) is higher than the number of studies (n=79). The 97 references [34-130] of the 79 included studies [52-130] can be found in Multimedia Appendix 2. Most studies (45/79, 57%) were conducted in North America (39/45, 87% in the United States and 6/45, 13% in Canada), followed by 9% (7/79) conducted only in the United Kingdom, 5% (4/79) only in the Republic of Korea, 5% (4/79) only in China (one of them was conducted in Hong Kong), 4% (3/79) only in the Netherlands (3/79, and 4% (3/79) only in Spain. 5% (4/79) of the studies were conducted in 2 or more countries, and 11% (9/79) were conducted in other single countries.

We used the MMAT [33] to assess the methodological quality of the included studies. No studies were excluded based on MMAT scores accordingly with the aim of our scoping review to identify and describe existing knowledge [26]. The MMAT scores of the 79 studies [52-130] can be consulted in Multimedia Appendix 3 [34-130]. A total of 58% (46/79) of the studies used an RCT design. Most RCTs (20/46, 43%) obtained a score of 4 stars, 26% (12/46) obtained a score of 3 stars, 24% (11/46) obtained a score of 2 stars, and 7% (3/46) obtained a score of 1 star. Per the study context, an RCT could not have 5 stars due to unfeasible patient blinding.

In Table 1, we structure our results according to 9 chronic disease clusters: diabetes (18/79, 23% of the studies), heart failure (13/79, 16% of the studies), other cardiovascular conditions (10/79, 13% of the studies), hypertension (8/79, 10% of the studies), multimorbidity and other combinations of chronic conditions (8/79, 10% of the studies), chronic obstructive pulmonary disease (COPD; 8/79, 10% of the studies), chronic pain (6/79, 8% of the studies), musculoskeletal conditions (6/79, 8% of the studies), and asthma (2/79, 3% of the studies).

We also structured our results according to the technologies used. The most frequent were wearable activity trackers (WATs; eg, a smartwatch or a device worn at the chest, waist built-in accelerometers, or connected pedometers). We found WATs in 53% (42/79) of the studies, BP monitors in 35% (28/79) of the studies, weighing scales in 25% (20/79) of the studies, BG monitors in 20% (16/79) of the studies, and electrocardiogram (ECG) monitors/devices in 10% (8/79) of the included studies. A total of 61% (48/79) of the intervention studies used a single wearable (or smart health) device, whereas 39% (31/79) of the studies used ≥2 devices.

In Table 2, we structure our results based on the types or combinations of wearables used. Table 2 provides an overview of the outcomes measured for each wearable or combination of wearables along with the reported effects. There are 2 points that require clarification. First, the study designs and sample sizes are not presented, but this information is available in Multimedia Appendix 3 [52-130]. Second, confounding factors such as multicomponent behavioral interventions are not considered. Full details of the sources used for Table 2 can also be found in Multimedia Appendix 3.

**Table 1:** Wearable devices per chronic disease category (N=79).

	WATs^a^ (n=42), n (%)	BP^b^ monitors (n=28), n (%)	WSs^c^ (n=20), n (%)	BG^d^ monitors (n=16), n (%)	ECG^e^ monitors (n=8), n (%)	Electronic medication tray or pillbox (n=4), n (%)	SpO_2_^f^ devices (n=4), n (%)	Photoplethysmography signal (n=2), n (%)	Inhaler adapter, indoor air quality monitor, portable spirometer, and a small device attached to inhalers (n=2), n (%)	Other (an ingestible sensor and a wearable patch with an accelerometer; n=1), n (%)
Diabetes (n=18)	12 (67)	2 (11)	2 (11)	10 (56)	0 (0)	1 (6)	0 (0)	1 (6)	0 (0)	0 (0)
Heart failure (n=13)	3 (23)	11 (85)	12 (92)	2 (15)	2 (15)	0 (0)	0 (0)	0 (0)	0 (0)	0 (0)
Other cardiovascular conditions (n=10)	5 (50)	5 (50)	3 (30)	0 (0)	6 (60)	0 (0)	0 (0)	0 (0)	0 (0)	0 (0)
Hypertension (n=8)	2 (25)	5 (62)	0 (0)	0 (0)	0 (0)	3 (38)	0 (0)	0 (0)	0 (0)	1 (12)
Multimorbidity and other combinations of chronic conditions (n=8)	3 (38)	4 (50)	2 (25)	4 (50)	0 (0)	0 (0)	0 (0)	0 (0)	0 (0)	0 (0)
COPD^g^ (n=8)	5 (62)	1 (12)	1 (12)	0 (0)	0 (0)	0 (0)	4 (50)	0 (0)	0 (0)	0 (0)
Chronic pain (n=6)	6 (100)	0 (0)	0 (0)	0 (0)	0 (0)	0 (0)	0 (0)	0 (0)	0 (0)	0 (0)
Musculoskeletal conditions (n=6)	5 (83)	0 (0)	0 (0)	0 (0)	0 (0)	0 (0)	0 (0)	1 (17)	0 (0)	0 (0)
Asthma (n=2)	1 (50)	0 (0)	0 (0)	0 (0)	0 (0)	0 (0)	0 (0)	0 (0)	2 (100)	0 (0)
Total studies	42 (53)	28 (35)	20 (25)	16 (20)	8 (10)	4 (5)	4 (5)	2 (3)	2 (3)	1 (1)

^a^WAT: wearable activity tracker.

^b^BP: blood pressure.

^c^WS: weighing scale.

^d^BG: blood glucose.

^e^ECG: electrocardiogram.

^f^SpO_2_: oxygen saturation.

^g^COPD: chronic obstructive pulmonary disease.

**Table 2:** Summary of wearables used, outcomes, and reported effects^a^.

Wearables	Clinical outcomes (eg, HbA_1c_^b^ or glucose levels, BP^c^, HR^d^, weight, BMI, sleep time or quality, and pain)	Behavioral outcomes (eg, PA^e^ levels, activation, motivation, self-efficacy, and knowledge)	Patient technology experience outcomes (eg, use or adherence, satisfaction, and engagement)	Holistic or biopsychosocial outcomes (eg, HRQoL^f^ and physical well-being)	Health care system outcomes (eg, use, effectiveness, and cost-effectiveness)
WATs^g^ only [52-82]	19 outcomes^h^ (BP [56], sleep time and quality [56], disability and pain [58]; BP [62], weight [62], waist circumference [62], BMI [62], BP [63], frailty [65], weight [65], BMI [65], disability at 6 months [67], pain [70], cardiorespiratory endurance [71], BMI [71], functional status [78], weight [79], insomnia severity [82], and acceptance of sleep difficulties [82]) 3 outcomes^i^ (Pain [55], disability at 12 months [67], and KOOS^j^, pain and symptoms [69]) 11 outcomes^k^ (severe COPD^l^ exacerbations [53], glucose level [62,66,71,80], weight [66], BMI [66], waist circumference [66], muscle strength [71,80], and cholesterol level [71])	13 outcomes^h^ (self-reported walking and PA goals [52], decreased depressive symptoms [56], PA [66], perceived walking habits [70], MVPA^m^ [70], daily step count [72,75,76], work productivity at 3 months [74], fatigue [76], heart failure knowledge [78], motivation to engage in behaviors [78], and minutes of daily PA [81]) 9 outcomes^i^ (PA levels [53], engagement levels [55], work productivity [55], PA [58], adherence to exercise guidelines [61], session attendance of a behavioral lifestyle intervention [65], mean MVPA time [69], mean daily steps and sedentary time [69], and KOOS, activities, sport, and recreation function [69]) 8 outcomes^k^ (self-management [52], functional activity capacity [53], self-efficacy [57], improvement in functioning and walking [64], work productivity at 6 and 12 months [74], self-efficacy to maintain PA [75], PA [79], and general self-efficacy [81]) 1 outcome^n^ (step count [57])	13 outcomes^i^ (patient experience or satisfaction with WATs [54,55,64-66,68,78,81], satisfaction with wearable motion sensors with straps [55], acceptability and usability [60], and acceptance of forms of recommendations [55,64,77]) 2 outcomes^n^ (some technical or dexterity-related difficulties [64], acceptability, and responses to [waist-worn] wearable vibration prompts [73])	5 outcomes^h^ (intervention group pretest-posttest health status [59,60], QoL^o^ [78], HRQoL [79], QoL and mental health score [81]) 1 outcome^i^ (KOOS and HRQoL [69]) 5 outcomes^k^ (HRQoL [53,72], health status changes between intervention and control groups [59,60], and QoL [65])	2 outcomes^h^ [60,78] (unplanned hospital visits [60] and heart failure–related hospitalization [78]) 6 outcomes^i^ (unplanned hospital visits and admissions during a 6-month follow-up period [59], hospital admissions during follow-up [60], continuity of care [60], incremental cost-effectiveness ratio [59,60], and person-centeredness [60])
Only BG^p^ monitoring devices (but with more outcomes measured than only glycemic control) [83-86]	4 outcomes^h^ (glucose level [83,84,86] and cholesterol control [84]) 2 outcomes^i^ (weight [83] and glucose level [85]) 5 outcomes^k^ (weight [84,86], BP [83,84], and cardiovascular risk [84])	2 outcomes^h^ (medication management [84] and knowledge about diabetes and BG testing [84]) 2 outcomes^k^ (skill and technique acquisition [85], depressive symptoms and lifestyle changes [85])	2 outcomes^h^ [84] (overall treatment satisfaction [84], willingness to recommend treatment to others [84])	1 outcome^k^ (HRQoL [85])	2 outcomes^h^ (initiating web messages to providers, mostly nurse care managers [84], health service navigation self-management [85]) 1 outcome^k^ (health care use [84])
WATs and BG monitoring devices [87,88]	1 outcome^h^ (glucose level [88])	1 outcome^h^ (self-care activity scores [88]) 1 outcome^i^ (self-regulation behaviors [87])	—^q^	—	—
WATs, BG monitoring devices, and connected WSs^r^ [89,90]	1 outcome^i^ (weight [90])	1 outcome^i^ (reduction of dose of oral hypoglycemic agents or insulin [90]) 2 outcomes^n^ (changes in caloric intake over time [90], mean daily step count [90])	1 outcome^i^ (patient experience and satisfaction [89])	—	—
BP monitoring devices only [91-94]	—	1 outcome^h^ (self-efficacy [93]) 1 outcome^i^ (self-efficacy [92]) 1 outcome^k^ (hypertension knowledge and participants’ perspectives on an mHealth^s^-based care model [91])	—	—	1 outcome^i^ (all-cause 30-day readmissions [93]) 1 outcome^k^ (ED^t^ visits [93])
WATs and BG and BP monitoring devices [95]	1 outcome^h^ (VO_2_max^u^ [95]) 2 outcomes^i^ (weight [95], waist circumference [95])	2 outcomes^i^ (PA [95] and adherence rates for self-monitoring [95])	1 outcome^i^ (patient experience and satisfaction [95])	—	—
BG and BP monitoring devices and medication tray or pillbox devices [96]; BP monitoring devices and medication pillbox or tray devices [97-99]	4 outcomes^h^ (glucose level [96], BP control [97,99], and resting BP [99]) 1 outcome^i^ (ambulatory BP [99])	2 outcomes^i^ (medication adherence [97,99])	1 outcome^i^ (patient experience and satisfaction [96])	—	—
BG and BP monitoring devices [100,101], WSs [102,103], and ECG^v^ devices [104]	1 outcome^h^ (BG and BP control [101]) 2 outcomes^i^ (weight, BP, BG, and symptoms reported by patients [102], BG, BP, and weight [103]) 1 outcome^k^ (body fat, BP, and BG [100])	1 outcome^h^ (changes in medication adherence, general adherence to treatment, adherence to disease-specific activities, and diabetes and hypertension knowledge [101]) 1 outcome^i^ (health self-management [104])	1 outcome^i^ (patient experience and satisfaction [102]) 1 outcome^k^ (patient experience and satisfaction [100])	—	1 outcome^k^ (health care resource use [104])
Connected WSs only [105]	2 outcomes^i^ (weight change [105], waist circumference, BP, and BG [105])	—	—	—	—

BP monitoring devices and WSs [106-113]	1 outcome^i^ (BNP^w^ values [112])	1 outcome^h^ (self-efficacy and self-care management [112]) 4 outcomes^i^ (self-efficacy and self-care management [107,108,113], daily monitoring after the intervention [108]) 1 outcome^k^ (the use of guideline-recommended medical therapy [110])	5 outcomes^i^ (usability and adherence to devices [106,108,111], end-user experience [107], contribution to a sense of safety and security after hospital discharge [107]) 1 outcome^m^ (use of the devices [109])	1 outcome^h^ (QoL [112]) 2 outcomes^i^ (QoL [107,113]) 1 outcome^k^ (physical well-being of participants [110])	2 outcomes^h^ (30-day all-cause readmission after discharge [109], health service use [112]) 3 outcomes^i^ (unscheduled ED revisits, readmission to hospital, and overall length of hospitalization [107], health system cost-effectiveness, including cost reduction and hospital bed capacity [107], proximity and communication with health care team or physicians [113]) 1 outcome^k^ (hospital resource use [113]) 1 outcome^n^ (traditional communication and engagement with providers prevailed, delaying access to care [108])
ECG devices only [114,115]	2 outcomes^h^ (detection of AF^x^, flutter recurrence [114], and detection of AF or other atrial arrhythmias [115])	1 outcome^h^ (two scales of the PCS^y^: Physical Functioning and Role Physical, and two scales of the MCS^z^: Vitality and Social Functioning [115])	1 outcome^i^ [114] (monitor usage among patients with AF [114])	1 outcome^h^ (two scales of the MCS: Role-Emotional and Mental Health [115]) 1 outcome^k^ (HRQoL in patients with AF [114])	—
WATs and ECG devices (a chest strap) [116]; WATs, ECG devices, and sleep trackers [117]	1 outcome^h^ (the eGFR^aa^ [117]) 1 outcome^k^ (body weight and BMI [117])	2 outcomes^h^ (self-care management and confidence [116], self-efficacy and self-management [117]) 2 outcomes^i^ (medication adherence [116], number of steps [117])	—	1 outcome^i^ [117] (QoL [117]) 1 outcome^n^ [116] (QoL [116])	—
ECG devices, BP monitoring devices, and WSs (a small sensor worn on the patient’s chest wall) [118]; ECG devices, BP monitoring devices, and WSs with [119,120] or without [121] WATs and sleep trackers	—	1 outcome^h^ (lifestyle behavior [120]) 1 outcome^i^ (self-care management and confidence [118])	2 outcomes^i^ (patient experience and satisfaction [118], adherence to mHealth program [121]) 1 outcome^k^ (patient experience and satisfaction [119])	1 outcome^k^ (QoL [120])	1 outcome^i^ (rehospitalization rates [118])
An ingestible sensor and a wearable patch that incorporates an accelerometer [122]	1 outcome^i^ (BP [122])	—	1 outcome^i^ (experiences with a digital health feedback system [122])	—	1 outcome^i^ (patients’ experiences with pharmacists [122])
COPD multicomponent systems (monitoring of SpO_2_^ab^) [123-126]; SpO_2_ device only [123]; inhaler adherence monitoring device+BG monitoring device [124]; SpO_2_ device+BP device+WS [125]; SpO_2_ device+WAT [126]	1 outcome^k^ (symptom scores [125])	3 outcomes^h^ (awareness level [123], self-efficacy [123], behavioral intention [123]) 1 outcome^i^ (self-management skills [125])	1 outcome^i^ (continuous wearing of a vest may be stressful [124])	1 outcome^h^ (between-group generic health status differences [126])	1 outcome^h^ (median number of visits to practice nurses [126]) 1 outcome^i^ (median number of visits to general practitioners [126]) 2 outcomes^k^ (hospitalizations, ED visits, or clinic visits [125,126])
Asthma devices (an inhaler adapter, an indoor air quality monitor, a portable spirometer, and a fraction exhaled nitric oxide device [127]; a small electronic medication monitor attached to inhalers [128])	2 outcomes^h^ (asthma control [127], exacerbations [127])	1 outcome^h^ (inhaler use [ICS^ac^ and SABAs^ad^] [128])	1 outcome^h^ (technology acceptance [127])	1 outcome^h^ (QoL [127])	—
Photoplethysmography signal devices [129,130]	3 outcomes^h^ (difference in the ASDAS^ae^ [130], total pain [130], VO_2_max [130]) 3 outcomes^i^ (glucose level [129], weight [129], and BP [129])	1 outcome^h^ (between-group frequency of difficulty feeling high motivation [130])	1 outcome^i^ [129] (patient experience and satisfaction [129])	—	—

^a^The results presented in this table can be interpreted as follows. Can we significantly improve clinical, behavioral, technology experience, psychosocial, and health care system effects or results by using a specific wearable or a combination of wearables? Taking the first row as an example, we identified 31 intervention studies in which only wearable activity trackers were used. In these 31 interventions, we identified 19 clinical, 13 behavioral, 5 holistic or biopsychosocial, and 2 health system outcomes showing positive effects or results (reported as statistically significant in the studies).

^b^HbA_1c_: glycated hemoglobin.

^c^BP: blood pressure.

^d^HR: heart rate.

^e^PA: physical activity.

^f^HRQoL: health-related quality of life.

^g^WAT: wearable activity tracker.

^h^Positive effect (identified as statistically significant).

^i^Neutral effect (positive effect but not identified as statistically significant).

^j^KOOS: Knee injury and Osteoarthritis Outcome Score.

^k^Neutral effect.

^l^COPD: chronic obstructive pulmonary disease.

^m^MVPA: moderate to vigorous PA.

^n^Negative effect.

^o^QoL: quality of life.

^p^BG: blood glucose.

^q^Not applicable.

^r^WS: weighing scale.

^s^mHealth: mobile health.

^t^ED: emergency department.

^u^VO_2_max: volume of oxygen maximum.

^v^ECG: electrocardiogram.

^w^BNP: B-type natriuretic peptide.

^x^AF: atrial fibrillation.

^y^PCS: physical component summary.

^z^MCS: mental component summary.

^aa^eGFR: estimated glomerular filtration rate.

^ab^SpO_2_: peripheral capillary oxygen saturation.

^ac^ICS: inhaled corticosteroids.

^ad^SABA: short-acting beta-agonist.

^ae^ASDAS: Ankylosing Spondylitis Disease Activity Score.

The most frequently reported clinical outcomes were BP control-related, in 23% (18/79) of studies [56,62,63,83,92, 93,95,97-101,103-105,112,122,129]. The other clinical outcomes most reported were weight or BMI changes in 13% (10/79) of studies. The most frequently reported behavioral outcomes were PA or lifestyle changes, including the number of steps and self-reported walking, in 27% (21/79) of studies [52,53,57,58,61,63-66,69,70,72,75, 76,78-81,90,95,117]. Other behavioral outcomes, such as self-efficacy (9/79, 11%) [53,57,75,81,92,93,107,117,123], and self-care management (6/79, 8%) [88,107,112,113,116,118], also appeared in a substantial number of studies. Adherence to treatment or recommendations (12/79, 15%) [61,65,78,94,95,97,99,101,106, 111,116,121] and engagement (6/79, 8%) [55,78,89, 108,113,115] are also frequently reported. Other commonly reported outcomes include satisfaction with technology (12/79, 15%) [55,64,66,68,75,78,81,84,96,100,102,118], and technology usability (6/79, 8%) [60,78,96,106,108,111]. QoL or health-related QoL are assessed in 19% (15/79) of studies [53,65,72,79,81,85,94,102,107,114-117,120,127], reflecting a broad focus on clinical, behavioral, technological experience, holistic, and biopsychosocial aspects.

In terms of positive results, BP control-related outcomes were reported as statistically significant in 8% (6/79) of studies [56,62,63, 93,97,98], and PA or lifestyle behavior changes in 10% (8/79) of studies [52,66,70,72,75,76,81,120]. Other behavioral outcomes, such as self-efficacy and self-care management enhancement, were reported as statistically significant in 5% (4/79) [112,116,117,123] of studies. Positive changes in weight or BMI were reported as statistically significant in 4% (3/79,) of studies [62,65,79] but demonstrated neutral or non-significant results in 9% (7/79) of studies [83,86,88,103,105,117,129]. Other outcomes, such as QoL or health-related QoL (8/79, 10%) [65,72,102,107,113,114, 117,120], technology usability (5/79, 6%) [60,96,106,108,111], and adherence to devices (4/79, 5%) [65,95,101,111], also demonstrated non-significant or neutral findings in those studies.

As shown in Table 3, we then counted the number of outcomes measured for each chronic disease cluster according to our 5 outcome categories. The results showed that clinical and behavioral outcomes were the most assessed and measured. Full details of the sources used for Table 3 are available in Multimedia Appendix 4 [52-130].

**Table 3:** Effects of outcomes measured for each chronic disease cluster across 5 outcome categories (N=79)^a^.

Effect		Diabetes (n=18), n	Heart failure (n=13), n	Other cardiovascular conditions (n=10), n	Hypertension (n=8), n	Multimorbidity and other combinations of chronic conditions (n=8), n	COPD^b^ (n=8), n	Chronic pain (n=6), n	Musculoskeletal conditions (n=6), n	Asthma (n=2), n	Total, n
**Clinical (eg, HbA_1c_^c^or glucose levels, BP^d^, HR^e^, weight, BMI, sleep time or quality, and pain)**											
	Positive effect (identified as statistically significant)	16	0	3	5	2	0	4	5	2	37
	Neutral (positive effect but not identified as statistically significant)	8	2	0	2	3	0	2	1	0	18
	Neutral	15	0	1	0	1	2	0	0	0	19
	Negative	0	0	0	0	0	0	0	0	0	0
**Behavioral (eg, physical activity levels, activation, motivation, self-efficacy, and knowledge)**											
	Positive effect (identified as statistically significant)	4	4	7	1	1	4	1	4	1	27
	Neutral (positive effect but not identified as statistically significant)	5	7	2	2	1	2	3	4	0	32
	Neutral	2	1	1	1	2	2	2	1	0	12
	Negative	2	0	0	0	0	0	0	0	0	2
**Patient technology experience (eg, use or adherence, satisfaction, and engagement)**											
	Positive effect (identified as statistically significant)	2	0	0	0	0	0	0	0	1	3
	Neutral (positive effect but not identified as statistically significant)	8	8	3	1	2	0	5	0	0	27
	Neutral	0	0	1	0	1	0	0	0	0	2
	Negative	0	1	0	0	0	2	1	0	0	4
**Holistic and biopsychosocial (eg, health-related quality of life or physical well-being)**											
	Positive effect (identified as statistically significant)	0	2	1	0	3	1	0	1	1	9
	Neutral (positive effect but not identified as statistically significant)	0	2	1	0	0	0	1	1	0	5
	Neutral	2	1	2	0	1	3	0	1	0	10
	Negative	0	1	0	0	0	0	0	0	0	1
**Health care system (eg, use, effectiveness, and cost-effectiveness)**											
	Positive effect (identified as statistically significant)	2	3	0	0	1	1	0	0	0	7
	Neutral (positive effect but not identified as statistically significant)	0	4	1	1	4	1	0	2	0	13
	Neutral	1	1	1	0	1	2	1	0	0	7
	Negative	0	1	0	0	0	0	0	0	0	1
**Total**											
	Positive effect (identified as statistically significant)	24	9	11	6	7	6	5	10	5	—^f^
	Neutral (positive effect but not identified as statistically significant)	22	23	7	6	12	3	11	8	0	—
	Neutral	20	3	6	1	6	9	3	2	0	—
	Negative	2	3	0	0	0	2	1	0	0	—

^a^The results presented in this table can be interpreted as follows. Can we significantly improve clinical, behavioral, technology experience, psychosocial, and health care system effects or results by using one or more wearables? Taking the diabetes column as an example, 16 clinical, 4 behavioral, 2 patient experience, and 2 health care system outcomes showed positive results (reported as statistically significant in the included studies). There are 2 points that need to be clarified. First, the number of participants and the duration of the studies were not considered. Second, confounding factors such as multicomponent behavioral interventions were not considered either.

^b^COPD: chronic obstructive pulmonary disease.

^c^HbA_1c_: glycated hemoglobin.

^d^BP: blood pressure.

^e^HR: heart rate.

^f^Not applicable.

The following sections present the outcomes assessed and measured, as well as the effects reported for each wearable or combination of wearables within each chronic disease cluster.

### Diabetes

We identified 23% (18/79) of the studies in which participants with diabetes were recruited [54,62,65,66,68,71,80,83-90,95, 96,129]. The complete table with details is available in Multimedia Appendix 4.

Participants with diabetes used a WAT in 67% (12/18) of the studies. We identified 22% (4/18) of the studies that included only a connected glucometer but measuring more outcomes than only glycemic or HbA_1c_ levels [83-86]. A total of 75% (3/4) of these studies reported statistically significant positive effects between groups on HbA_1c_ or glycemic control at 6 [83,86] and 12 months [84]. In the remaining study, HbA_1c_ levels decreased in all groups but did not differ between groups [85]. WATs were used in 67% (12/18) of all diabetes studies and in 86% (12/14) of diabetes studies that did not use a connected glucometer. With only using WAT without BG monitoring, no effects were found on glucose or HbA_1c_ levels [62,66,71,80].

Across all 23% (18/79) of the diabetes studies included, positive effects reported as statistically significant were found for 16 clinical (glucose or HbA_1c_ levels [83,84,86], cholesterol control [84], maximal oxygen consumption [95], frailty [65], weight [62,65], BMI [62,65,71], cardiorespiratory endurance [71], waist-to-hip ratio circumference [71], and BP [62]), 4 behavioral (physical activity [66], self-care activity scores [88], medication management [84], and knowledge about diabetes [84]), 2 patient technology experience (overall treatment satisfaction [84] and willingness to recommend to others [84]), and 2 health care system (initiating web-based messages to providers [84] and health service navigation self-management [85]) outcomes. No holistic or biopsychosocial outcomes showed effects. In total, 2 weak negative behavioral effects were found on a small single-group study of 9 participants in which changes in caloric intake and mean daily step count declined over the 12-week intervention [90].

### Heart Failure

We identified 16% (13/79) of the studies in which participants with heart failure were recruited [61,78,102,106-113,116,118]. The complete table with details is available in Multimedia Appendix 4.

Most heart failure studies used connected weighing scales (12/13, 92%) and BP monitoring devices (11/13, 85%). WATs were used in 23% (3/13) of the studies [61,78,116]. Clinical outcomes were measured in 15% (2/13) of the studies [102,112], and both studies showed no effects. Positive effects reported as statistically significant were found for 4 behavioral (self-efficacy or self-care management [112,116], heart failure knowledge [78], and motivation to engage in behaviors [78]), 2 holistic or biopsychosocial (QoL [78,112]), and 3 health system (30-day all-cause readmission after discharge [109], health service use [112], and heart failure–related hospitalization [78]) outcomes. A total of 3 outcomes showed weak negative effects related to technology experience (ie, use of the devices [109]), holistic or biopsychosocial (ie, QoL related to wearing a chest strap [116]), and health care system (ie, delaying accessibility to care as traditional communication with providers prevailed [108]) outcomes.

### Other Cardiovascular Conditions

We grouped 13% (10/79) of the studies in which participants with cardiovascular diseases such as chronic heart disease, coronary artery disease, atrial fibrillation or atrial flutter, poststroke control or cardiac rehabilitation, acute myocardial infarction, and any combination of heart failure and hypertension were recruited [75,76,92,93,114,115,117,119-121]. The complete table with details is available in Multimedia Appendix 4.

ECG devices were used in 60% (6/10) of the studies, WATs and BP monitors were used in 50% (5/10) of the studies, and connected weighing scales were used in 30% (3/10) of the studies. Positive effects reported as statistically significant were found for 3 clinical (detection of atrial fibrillation or atrial flutter recurrence [114], detection of other atrial arrhythmias [115], and slower decline in the estimated glomerular filtration rate [117]) outcomes in which ECG devices were involved. Positive effects reported as statistically significant were found for 6 behavioral (daily step count or walking time [75,76], fatigue [76], self-efficacy or self-management [93,117], physical functioning [115], and lifestyle behavior [120]) outcomes. Positive effects were found for 1 holistic or biopsychosocial outcome (mental health domain scores [115]). All other outcomes had weak positive or neutral effects.

### Hypertension

We identified 10% (8/79) of the studies in which participants with hypertension were recruited [56,63,91,94,97-99,122]. The complete table with details is available in Multimedia Appendix 4.

A total of 62% (5/8) of the hypertension studies used BP monitors, 38% (3/8) used a connected medication tray or pillbox, and 25% (2/8) used WATs. Positive effects reported as statistically significant were found for 5 clinical (BP measures or control [56,63,97,99] and sleep time or quality [5]) and 1 behavioral (decreased depressive symptoms [56]) outcomes. No other patient technology experience, holistic and biopsychosocial, or health care system outcomes showed positive effects.

### Multimorbidity and Other Combinations of Chronic Conditions

We identified 10% (8/79) of the studies that recruited participants with ≥2 chronic conditions [60,79,81,100,101,103-105]. The complete table with details is available in Multimedia Appendix 4.

A total of 50% (4/8) of the studies used BG and BP monitoring devices [100,101,103,104], 38% (3/8) used only WATs [60,79,81], 25% (2/8) used connected weighing scales [103,105], and 12% (1/8) used ECG devices [104]. Positive effects reported as statistically significant in the studies were found for 2 clinical (weight [79] and BG and BP [101]), 2 behavioral (minutes of daily physical activity [81] and hypertension knowledge [101]), 3 holistic or biopsychosocial (health status [60], health-related QoL [79], and QoL and mental health score [81]), and 1 health care system (unplanned hospital visits [60]) outcomes. The wearable devices used had only weak positive or neutral effects on patient technology experience outcomes.

### Studies on COPD

We identified 10% (8/79) of the studies in which participants with COPD were recruited [53,57,72,73,123-126]. The complete table with details is available in Multimedia Appendix 4.

We identified a first group of 50% (4/8) of the studies comprising multicomponent systems in which oxygen saturation or inhaler adherence was monitored [123-126] and a second group of 50% (4/8) of the studies that used only WATs [53,57,72,73].

In the first group of 50% (4/8) of the studies, 1 clinical effect was measured and found out to be neutral (symptom scores [125]). However, positive effects reported as statistically significant were found for 3 behavioral (awareness level, self-efficacy, and behavioral intention [123]), 1 holistic or psychosocial (generic health status [126]), and 1 health care system (reduction of median number of visits to practice nurses [126]) outcomes. Negative effects were found for 1 patient technology experience outcome (stress related to continuously wearing a monitoring vest [126]).

In the second group of 50% (4/8) of the studies, which used only WATs, 1 clinical effect was measured and found out to be neutral (severe COPD exacerbations [53]). Positive effects reported as statistically significant were found for 1 behavioral outcome (daily step count [72]), whereas step count decreased over time in another study [57]. Another negative effect was reported for 1 patient technology experience outcome (low acceptability and responses to the waist-worn wearable vibration prompts [73]).

### Chronic Pain

We identified 8% (6/79) of the studies in which participants with chronic pain were recruited [52,55,58,64,67,77]. The complete table with details is available in Multimedia Appendix 4.

Only WATs were used in the identified studies on chronic pain. Positive effects reported as statistically significant were found for 4 clinical outcomes (symptom score [58], disability [58], pain [58], and chronic back pain–related disability at 6 months [67]). Another positive effect was found for 1 behavioral outcome (self-reported walking and physical activity goals [52]). All other outcomes had neutral or weak and nonsignificant positive effects, and negative effects were found on 1 patient technology experience outcome (technical or dexterity-related difficulties [64]).

### Musculoskeletal Conditions

We identified 8% (6/79) of the studies in which participants with osteoporosis, rheumatoid arthritis, systemic lupus erythematosus, or ankylosing spondylitis were recruited [59,69,70,74,82,130]. The complete table with details is available in Multimedia Appendix 4.

We identified 83% (5/6) of the studies on osteoporosis, rheumatoid arthritis, or systemic lupus erythematosus that all used WATs [59,69,70,74,82]. The remaining study recruited participants with ankylosing spondylitis, and their intervention used photoplethysmography signal technology [130]. Positive effects reported as statistically significant in this study were found for 3 clinical (Ankylosing Spondylitis Disease Activity Score [130]; total pain, fatigue, spinal pain, and morning stiffness intensity [130]; and maximal oxygen consumption back extensor endurance test and range of motion of cervical lateral flexion [130]) and 1 behavioral (frequency of difficulty with high motivation [130]) outcomes. Concerning the other 83% (5/6) of the studies, positive effects reported as statistically significant were found for 2 clinical (pain reduction [70] and Insomnia Severity Index score and acceptance of sleep difficulties [82]) and 3 behavioral (perceived walking habits [70], moderate to vigorous physical activity for participants with rheumatoid arthritis [70], and work productivity at the 3-month follow-up [74]) outcomes. Regarding the latter work productivity behavioral outcome, we noticed that the effects were no longer significant at the 6- and 12-month follow-ups [74].

### Asthma

We identified 3% (2/79) of the studies in which participants with asthma were recruited [127,128]. The complete table with details is available in Multimedia Appendix 4. Positive effects reported as statistically significant were found for 2 clinical (asthma control [127] and asthma exacerbations [127]), 1 behavioral (inhaler use [128]), 1 patient technology experience (technology acceptance [127]), and 1 holistic or biopsychosocial (QoL [127]) outcomes. Health care system outcomes were not investigated.

## Discussion

### Principal Findings

This scoping review aimed to achieve 3 main objectives related to health intervention studies involving wearable devices. The primary strength of this review lies in our broad portrayal of interventions across multiple chronic diseases, various wearable devices, and distinct categories of health and health care outcomes, along with their reported effects (positive, neutral, or negative). We selected these broad objectives because, as other reviews have highlighted [24,25], providing a comprehensive overview of this topic is relevant for informing and shaping future research directions.

First, we identified and described clusters of chronic diseases that have been studied. We observed significant disparities—diabetes and heart failure have been extensively researched, whereas musculoskeletal conditions and asthma have been studied much less frequently despite showing promising results. We intentionally excluded studies that solely involved BG monitoring devices measuring BG levels as the only outcome given the existing evidence [8-15]. Nevertheless, diabetes emerged as the most represented chronic disease, appearing in 23% (18/79) of the studies, whereas only 3% (2/79) of the studies included participants with asthma.

Second, we identified and described the technologies used. WATs were commonly used either alone or in conjunction with other devices tailored to specific chronic conditions, such as BG monitors, BP devices, ECG devices, and connected weighing scales. There was notable variation in technology use—while WATs were widely used, other devices such as oxygen saturation monitors, photoplethysmography sensors, inhaler adapters, indoor air quality monitors, portable spirometers, small devices attached to inhalers, ingestible sensors, and wearable patches with accelerometers were less common.

Third, we identified and described the outcomes measured in 5 distinct categories. Clinical and behavioral outcomes were the most frequently assessed, whereas patient technology experience, health care system (such as use or efficiency), and holistic or biopsychosocial (such as QoL) outcomes were reported less frequently.

### Limitations

This scoping review has several limitations. We did not integrate the individual interventions into our analysis. Although we report the measured outcomes, these results must be interpreted with caution as effect sizes were not considered. However, informed readers can consult the appended files that contain all the relevant information, including study design, sample size, intervention and participant characteristics, outcomes measured, and effects. We only conducted searches in the MEDLINE (Ovid) and Web of Science (Science Citation Index Expanded, Social Sciences Citation Index, Arts and Humanities Citation Index, and Emerging Sources Citation Index) databases, which may have led to missing sources. Confounding factors such as multicomponent behavioral strategy interventions were also not considered. Nevertheless, these confounding factors were similarly not addressed in a previous systematic review and meta-analysis on the effectiveness of WATs due to the heterogeneity of study designs and interventions [18]. In total, 3 reviewers extracted and assessed the quality of all the studies only once, although this was mitigated by data validation from an experienced researcher.

### Comparison With Other Reviews

Our findings revealed significant disparities in the chronic diseases studied. Type 2 diabetes and heart failure were extensively researched, whereas conditions such as musculoskeletal disorders, chronic pain, and asthma were less represented. This both complements and contrasts with the findings of another review, which noted type 2 diabetes, Parkinson disease, and chronic lower back pain as among the most studied conditions [25]. Many other reviews have focused on diabetes [8-15,21] and cardiovascular diseases [22,23]. One reason for this emphasis on cardiovascular disease and type 2 diabetes may be the inherent capabilities of wearables to monitor and manage these conditions [4], along with their availability and affordability. For instance, regarding asthma, we found promising effects on health outcomes but identified only a limited number of studies [127,128].

In terms of the technologies used, we observed that the most common devices used were WATs. This aligns with the number of reviews focusing on the effects of WATs for different diseases [16-20], often in combination with disease-specific devices. This observation is also consistent with those of other reviews as wearable technologies have emerged as promising tools for managing chronic diseases, offering continuous monitoring of vital signs, physical activity, and disease-specific markers [132,133]. Similarly to our findings, these devices have shown potential across various conditions, including BP and ECG monitors for cardiovascular diseases [22,23] and BG monitoring devices for diabetes [8,9,11], by enabling early detection of complications and promoting patient engagement [25,132-134].

Regarding outcomes, we identified 5 distinct categories in our review, with clinical and behavioral outcomes being the most frequently reported. This aligns with the growing body of research highlighting the role of wearables in monitoring physiological parameters and behaviors [134]. Similarly to our findings, other reviews have reported clinical outcomes such as glycemic control [8-15] as well as behavioral outcomes, including physical activity levels [16-20], medication adherence, and self-management [5,18].

### Future Directions

Despite the promising health outcomes reported, other reviews have highlighted that the quality of evidence varies considerably, with many studies limited by small sample sizes or short durations [25]. Researchers emphasize the need for more long-term studies and systematic reviews that incorporate RCTs with larger sample sizes to measure the effectiveness of wearables across a wider variety of chronic diseases [18,24,25]. There is a need for rigorous, long-term studies to establish the clinical effectiveness and cost-efficiency of wearables in chronic disease self-management [25,132].

Our findings indicate that patient technology experience, holistic and biopsychosocial, and health care system outcomes have been covered less and could benefit from more robust studies. As the integration of wearable technology into health research and clinical practice continues to expand, establishing comprehensive guidelines to ensure effective use across diverse chronic conditions and applications will be important, ranging from chronic disease management to real-time health monitoring for specific or underrepresented populations [134].

In line with our findings, which show that health care system outcomes are reported less frequently than clinical and behavioral outcomes and often have inconclusive effects, a significant challenge will be integrating wearable data into existing health care systems and clinical workflows [135].

### Conclusions

This review provides a comprehensive overview of wearable devices used in chronic disease self-management intervention studies, revealing disparities in both the range of chronic diseases studied and the variety of wearable devices used. We described the clinical and behavioral benefits of wearable devices, particularly for activity trackers, BG and BP monitors, and ECG wearable devices. These findings lay a foundation for future research, offering researchers valuable insights to further explore health care system outcomes, validate the impact of concomitant device use, and expand their use to other chronic diseases.

## Appendix

Multimedia Appendix 1 Database search strategies.

Multimedia Appendix 2 Characteristics of the sources of evidence.

Multimedia Appendix 3 Characteristics of the studies with Mixed Methods Appraisal Tool scores.

Multimedia Appendix 4 Connected wearable devices used, outcomes measured, and effects per chronic disease cluster.

Multimedia Appendix 5 The PRISMA-ScR (Preferred Reporting Items for Systematic Reviews and Meta-Analyses extension for Scoping Reviews) checklist.

## Abbreviations

BG: blood glucose
BP: blood pressure
COPD: chronic obstructive pulmonary disease
ECG: electrocardiogram
HbA1c: glycated hemoglobin
MMAT: Mixed Methods Appraisal Tool
PRISMA: Preferred Reporting Items for Systematic Reviews and Meta-Analyses
PRISMA-ScR: Preferred Reporting Items for Systematic Reviews and Meta-Analyses extension for Scoping Reviews
QoL: quality of life
RCT: randomized controlled trial
WAT: wearable activity tracker

## Data Availability

The PRISMA-ScR (Preferred Reporting Items for Systematic Reviews and Meta-Analyses extension for Scoping Reviews) checklist [27] was included in the manuscript submission and can be consulted in Multimedia Appendix 5. The data sets generated and analyzed during this study are available from the corresponding author (MPG) on reasonable request.
