# Supplementary material for: Wearable Devices for Supporting Chronic Disease Self-Management: Scoping Review
Source: Interact J Med Res. 2024 Dec 9;13:e55925. doi: 10.2196/55925 (PMC11667132; doi:10.2196/55925)
Supplement: Multimedia Appendix 1 [file ijmr_v13i1e55925_app1.pdf]

Database search strategies.

Medline (Ovid)
Date of the search: 30-06-2022
Database limit: 2012 -Current

#	Search strategy	Results
1	exp Chronic Disease/	595614
2	((chronic* or longterm or long-term or longstanding or long-standing or ongoing or persisten*) adj2 (comorbid* or co-morbid* or condition* or disabilit* or disease* or disorder* or ill or illness* or morbidit* or syndrom* or symptom*)).tw,kf.	388982
3	1 or 2	868247
4	exp Arthritis/	285103
5	(arthriti* or arthros#s or osteoarthriti* or osteo-arthriti* or osteoarthros#s or osteo-arthros#s or polyarthriti* or poly-arthriti* or spondylarthriti* or spondyl-arthriti*).tw,kf.	279696
6	exp Asthma/	138157
7	asthma*.tw,kf.	172280
8	Chronic Pain/	20156
9	Pain, Intractable/	6331
10	((chronic* or constant* or continual* or continuous* or ceaseless* or endless* or incessant* or interminabl* or intractable or longterm or long-term or nonstop* or non-stop* or perpetual* or persist* or recur* or refractory or sustained or relentless* or unabat* or unceasing or unending or uninterrupt* or unrelenting or unrelieved or unremitt*) adj3 (neuralgi* or neuropath* or pain*)).tw,kf.	111023
11	exp Neoplasms/	3705160
12	(neoplas* or cancer* or tumour* or tumor* or carcinoma* or malignan* or metasta* or oncolog*).tw,kf.	3827452
13	(adenoma* or adenocarcinoma*or adeno-carcinoma* or blastoma* or carcinosarcoma* or carcino-sarcoma* or hematoma* or hepatoblastoma* or hepato-blastoma* or leukemia* or leukaemia* or lymphoma*or melanoma*or mesenchymoma* or mesothelioma* or sarcoma* or thymoma*).tw,kf.	551614
14	Renal Insufficiency, Chronic/	31923
15	(chronic adj2 (renal or kidney*) adj3 (condition* or disease* or disorder* or insufficien*)).tw,kf.	74391
16	(CKD or CKDs).tw,kf.	37647
17	Diabetes Mellitus/	131390
18	Diabetes Mellitus, Type 1/	82951
19	Diabetes Mellitus, Type 2/	158888
20	Latent Autoimmune Diabetes in Adults/	144
21	diabet*.tw,kf.	719525
22	(("Type 1" or "Type I" or "Type 2" or "Type II" or ID or NID) adj DM).tw,kf.	4966
23	(IDDM or NIDDM).tw,kf.	12647

24	exp Dyslipidemias/	85153
25	(dyslipidemi* or dyslipoproteinemi* or dyslipo-proteinemi* or hyperlipemi* or hyperlipid?emi* or hyper-lipemi* or hyper-lipid?emi* or lipemi* or lipid?emi* or hypercholester?emi* or hypercholester?emi* or hypertriglyceridemi* or hyper-triglyceridemi*).tw,kf.	85158
26	exp Inflammatory Bowel Diseases/	92079
27	(inflam* adj1 (bowel* or colon*)).tw,kf.	61306
28	IBD.tw,kf.	31031
29	(Crohn or "Crohn's").tw,kf.	53000
30	(ileitis adj2 (regional or terminal)).tw,kf.	1312
31	(Ileocolitis or ileo-colitis).tw,kf.	470
32	((proctocolitis or procto-colitis) adj2 idiopathic).tw,kf.	38
33	((colorectitis or proctocolitis or procto-colitis) adj2 (ulcer* or mucosal)).tw,kf.	69
34	colitis gravis.tw,kf.	5
35	exp Pulmonary Disease, Chronic Obstructive/	63834
36	(chronic adj2 obstructi* adj3 (pulmonary or airway* or air way* or lung or lungs or airflow* or air flow* or bronchitis or bronchopulmonar* or broncho-pulmonar*)).tw,kf.	63460
37	(emphysema? adj3 (pulmonary or airway* or air way* or lung or lungs or airflow* or air flow* or centriacinar or centri-acinar or centrilobular or centri-lobular or panacinar or pan-acinar or panlobular or pan-lobular)).tw,kf.	10146
38	(COPD or COAD).tw,kf.	54585
39	exp Cardiovascular Diseases/	2629418
40	((artery or arteries or arterial or atrial* or atrium* or cardiac* or cardio* or cardiovascul* or cardio-vascul* or coronary or heart* or myocard* or ventricle*) adj3 (arrest* or aneurysm* or arrhythmia* or arrythmia* or disease* or disorder* or dysrhythmia* or dysrythmia* or dysfunction* or failure* or fibrillation* or ischemi* or infarction* or obstruct* or occlu* or rupture* or stenos#s or syndrom* or thrombo* or vasospas* or vaso-spas*)).tw,kf.	1195038
41	(arterioscleros* or arterio-scleros* or atheroscleros* or athero-scleros*).tw,kf.	143403
42	hypertens*.tw,kf.	481990
43	((elevated or high*) adj2 blood pressure*).tw,kf.	36948
44	or/4-43	8935364
45	3 or 44	9337777
46	("Self Care" or "Self Management" or "self administration" or "self medication*" or "self monitoring" or "self testing").kf,tw.	66434
47	exp Self Care/	60270
48	46 or 47	99926
49	(connect* adj3 (device* or technolog* or health)).kf,tw.	5612
50	(e-health or ehealth or "electronic health" or m-health or mHealth or "mobile health").kf,tw.	43599

51	((Wearable adj2 (Technolog* or device*)) or Accelerometer* or "Step count*" or Pedometer* or "fitness tracker*" or (activit* adj2 monitor*) or Fitbit or "Apple Watch" or Garmin or smartwatch* or "smart watch*").kf,tw.	41466
52	exp Wearable Electronic Devices/	16996
53	("internet of things" or iot).kf,tw.	5799
54	Internet of Things/	722
55	sensor*.kf,tw.	422619
56	or/49-55	516274
57	45 and 48 and 56	4016
58	limit 57 to yr="2012 -Current"	3533

Web of Science (A&HCI , ESCI , CPCI-SSH , CPCI-S , SCI-EXPANDED , SSCI)

Date of the search: 30-06-2022

Database limit: 2012 -Current

#	Search strategy	Results
1	TS=((chronic* or longterm or long-term or longstanding or long-standing or ongoing or persisten*) NEAR/2 (comorbid* or co-morbid* or condition* or disabilit* or disease* or disorder* or ill or illness* or morbidit* or syndrom* or symptom*))	552 860
2	TS=(arthriti* or arthros?s or osteoarthriti* or osteo-arthriti* or osteoarthros?s or osteo-arthros?s or polyarthriti* or poly-arthriti* or spondylarthriti* or spondyl-arthriti*)	402 136
3	TS=(asthma*)	228 433
4	TS=((chronic* or constant* or continual* or continuous* or ceaseless* or endless* or incessant* or interminabl* or intractable or longterm or long-term or nonstop* or non-stop* or perpetual* or persist* or recur* or refractory or sustained or relentless* or unabat* or unceasing or unending or uninterrupt* or unrelenting or unrelieved or unremitt*) NEAR/3 (neuralgi* or neuropath* or pain*))	129 799
5	TS=(neoplas* or cancer* or tumour* or tumor* or carcinoma* or malignan* or metasta* or oncolog*)	4 781 922
6	TS=(adenoma* or adenocarcinoma*or adeno-carcinoma* or blastoma* or carcinosarcoma* or carcino-sarcoma* or hematoma* or hepatoblastoma* or hepato-blastoma* or leukemia* or leukaemia* or lymphoma*or melanoma*or mesenchymoma* or mesothelioma* or sarcoma* or thymoma*)	698 717
7	TS=((chronic NEAR/2 (renal or kidney*) NEAR/3 (condition* or disease* or disorder* or insufficien*)))	99 266
8	TS=(CKD or CKDs)	41 756
9	TS=(diabet*)	898 189
10	TS=(("Type 1" or "Type I" or "Type 2" or "Type II" or ID or NID) NEAR/1 DM)	6 291
11	TS=(IDDM or NIDDM)	22 182
12	TS=(inflam* NEAR/1 (bowel* or colon*))	101 179
13	TS=(Crohn or "Crohn's")	60 138

14	TS=(ileitis NEAR/2 (regional or terminal))	587
15	TS=(Ileocolitis or ileo-colitis)	367
16	TS=((proctocolitis or procto-colitis) NEAR/2 idiopathic)	16
17	TS=((colorectitis or proctocolitis or procto-colitis) NEAR/2 (ulcer* or mucosal))	48
18	TS=("colitis gravis")	3
19	TS=(chronic NEAR/2 structi* NEAR/3 (pulmonary or airway* or "air way*" or lung or lungs or airflow* or "air flow*" or bronchitis or bronchopulmonar* or broncho-pulmonar*))	4
20	TS=(emphysema$ NEAR/3 (pulmonary or airway* or "air way*" or lung or lungs or airflow* or "air flow*" or centriacinar or centri-acinar or centrilobular or centri-lobular or panacinar or pan-acinar or panlobular or pan-lobular))	8 386
21	TS=(COPD or COAD)	77 894
22	TS=(dyslipidemi* or dyslipoproteinemi* or dyslipo-proteinemi* or hyperlipemi* or hyperlipid$emi* or hyper-lipemi* or hyper-lipid$emi* or lipemi* or lipid$emi* or hypercholester$emi* or hypercholester$emi* or hypertriglyceridemi* or hyper-triglyceridemi*)	95 584
23	TS=((artery or arteries or arterial or atrial* or atrium* or cardiac* or cardio* or cardiovascul* or cardio-vascul* or coronary or heart* or myocard* or ventricle*) NEAR/3 (arrest* or aneurysm* or arrhythmia* or arrythmia* or disease* or disorder* or dysrhythmia* or dysrythmia* or dysfunction* or failure* or fibrillation* or ischemi* or infarction* or obstruct* or occlu* or rupture* or stenos?s or syndrom* or thrombo* or vasospas* or vaso-spas*))	1 550 536
24	TS=(arterioscleros* or arterio-scleros* or atheroscleros* or athero-scleros*)	209 524
25	TS=(hypertens*)	595 988
26	TS=((elevated or high*) NEAR/2 "blood pressure*")	37 740
27	#26 OR #25 OR #24 OR #23 OR #22 OR #21 OR #20 OR #19 OR #18 OR #17 OR #16 OR #15 OR #14 OR #13 OR #12 OR #11 OR #10 OR #9 OR #8 OR #7 OR #6 OR #5 OR #4 OR #3 OR #2 OR #1	8 759 487
28	TS=("Self Care" or "Self Management" or "self administration" or "self medication*" or "self monitoring" or "self testing")	91 022
29	TS=(connect* NEAR/3 (device* or technolog* or health))	30 189
30	TS=(e-health or ehealth or "electronic health" or m-health or mHealth or "mobile health")	57 163
31	TS=((Wearable NEAR/2 (Technolog* or device*)) or Accelerometer* or "Step count*" or Pedometer* or "fitness tracker*" or (activit* NEAR/2 monitor*) or Fitbit or "Apple Watch" or Garmin or smartwatch* or "smart watch*")	106 207
32	TS=("internet of things" or iot)	93 670
33	S=(sensor*)	1 365 082
34	#32 OR #33 OR #31 OR #30 OR #29	1 575 188
35	#34 AND #28 AND #27 (A&HCI, ESCI, CPCI-SSH, CPCI-S, SCI-EXPANDED, SSCI)	3 528
36	#34 AND #28 AND #27 (A&HCI, ESCI, SCI-EXPANDED, SSCI)	3 251

37	#34 AND #28 AND #27 (A&HCI , ESCI, SCI-EXPANDED, SSCI)<br>Timespan: 2012-01-01 to 2022-12-31 (Index Date)	2 994
