# Supplementary material for: Wearable Devices for Supporting Chronic Disease Self-Management: Scoping Review
Source: Interact J Med Res. 2024 Dec 9;13:e55925. doi: 10.2196/55925 (PMC11667132; doi:10.2196/55925)
Supplement: Multimedia Appendix 2 [file ijmr_v13i1e55925_app2.pdf]

Characteristics of the sources of evidence.

References	Author et al., Year	Country or Countries	Title(s)	Chronic disease(s)
[123]	Alharbey & Chatterjee, 2019	USA	An mHealth Assistive System "MyLung" to Empower Patients with Chronic Obstructive Pulmonary Disease: Design Science Research	Chronic obstructive pulmonary disease (COPD)
[102]	Alnosayan et al., 2017	USA	Design and Usability of a Heart Failure mHealth System: A Pilot Study	Heart failure (HF)
[52]	Amorim et al., 2019	Australia	Integrating Mobile-health, health coaching, and physical activity to reduce the burden of chronic low back pain trial (IMPACT): a pilot randomised controlled trial	Chronic low back pain
[119]	Andersen et al., 2020	Denmark	Experiences With Wearable Activity Data During Self-Care by Chronic Heart Patients: Qualitative Study	Chronic heart disease
[44,53]	Arbillaga-Etxarri et al., 2018<br>Koreny et al., 2019	Spain	(RCT): Long-term efficacy and effectiveness of a behavioural and community-based exercise intervention (Urban Training) to increase physical activity in patients with COPD: a randomised controlled trial<br><br>(Cohort study): Determinants of study completion and response to a 12-month behavioral physical activity intervention in chronic obstructive pulmonary disease: A cohort study	Chronic obstructive pulmonary disease (COPD)
[54]	Arsand et al., 2015	Czech Republic and Norway	Performance of the first combined smartwatch and smartphone diabetes diary application study	Type 1 diabetes
[116]	Athilingam et al., 2017	USA	A Mobile Health Intervention to Improve Self-Care in Patients With Heart Failure: Pilot Randomized Control Trial	Heart failure (HF)
[55]	Bailey et al., 2020	USA	Digital Care for Chronic Musculoskeletal Pain: 10,000 Participant Longitudinal Cohort Study	Chronic knee and back pain
[56]	Baron et al., 2019	USA	Technology Assisted Behavior Intervention to Extend Sleep Among Adults With Short Sleep Duration and Prehypertension/Stage 1 Hypertension: A Randomized Pilot Feasibility Study	Prehypertension/Stage 1 Hypertension
[105]	Bennett et al., 2018	USA	Effectiveness of an App and Provider Counseling for Obesity Treatment in Primary Care	(Hypertension, diabetes and hyperlipidemia) and obesity
[57]	Bentley et al., 2020	United Kingdom	The Use of a Smartphone App and an Activity Tracker to Promote Physical Activity in the Management of Chronic Obstructive Pulmonary Disease: Randomized Controlled Feasibility Study	Chronic obstructive pulmonary disease (COPD)
[38,104]	Bloss et al., 2016<br>Kim et al., 2016	USA	RCT: Bloss et al., 2016<br>A prospective randomized trial examining health care utilization in individuals using multiple smartphone-enabled biosensors	Hypertension, insulin-dependent or non-insulin

References	Author et al., Year	Country or Countries	Title(s)	Chronic disease(s)
			Sub study: Kim et al., 2016<br>The Influence of Wireless Self-Monitoring Program on the Relationship Between Patient Activation and Health Behaviors, Medication Adherence, and Blood Pressure Levels in Hypertensive Patients: A sub study of a Randomized Controlled Trial	dependent diabetes, and/or with arrhythmia
[120]	Broers et al., 2020	Spain and the Netherlands	Personalized eHealth Program for Life-style Change: Results From the "Do Cardiac Health Advanced New Generated Ecosystem (Do CHANGE 2)" Randomized Controlled Trial	Cardiac patients (heart failure, coronary artery disease, hypertension)
[97]	Chandler et al., 2019	USA	Impact of a Culturally Tailored mHealth Medication Regimen Self-Management Program upon Blood Pressure among Hypertensive Hispanic Adults	Hypertension
[58]	Chhabra et al., 2018	India	Smartphone app in self-management of chronic low back pain: a randomized controlled trial	Chronic low back pain
[59]	Colomina et al., 2021	Spain	Implementing mHealth-Enabled Integrated Care for Complex Chronic Patients with Osteoarthritis Undergoing Primary Hip or Knee Arthroplasty: Prospective, Two-Arm, Parallel Trial	Complex Chronic Patients with Osteoarthritis Undergoing Primary Hip or Knee Arthroplasty
[91]	Culhane-Pera et al., 2022	USA	Mobile health technology for hypertension management with Hmong and Latino adults: mixed-methods community-based participatory research	Hypertension
[118]	Dadosky et al., 2018	USA	Telemanagement of Heart Failure Patients Across the Post-Acute Care Continuum	Heart failure (HF)
[98]	Davidson et al., 2015	USA	Evaluation of an mHealth Medication Regimen Self-Management Program for African American and Hispanic Uncontrolled Hypertensives	Hypertension
[34,60]	de Batlle et al., 2020<br>de Battle et al., 2021	Spain	Implementing Mobile Health–Enabled Integrated Care for Complex Chronic Patients: Patients and Professionals' Acceptability Study (2020)<br><br>Implementing Mobile Health–Enabled Integrated Care for Complex Chronic Patients: Intervention Effectiveness and Cost-Effectiveness Study (2021)<br>*N.B. The 2021 cost-effectiveness study take into account only the use case # 1 participants (UC1)": "home-dwelling patients, with chronic conditions and a history of visits to the emergency room (ER) leading to hospitalizations".)*	2 different use cases (UCs): a history of hospitalizations for chronic obstructive pulmonary disease or heart failure (use case [UC] 1), or a scheduled major elective hip or knee arthroplasty (use case [UC] 2)
[61]	Deka et al., 2019	USA	MOVE-HF: an internet-based pilot study to improve adherence to exercise in patients with heart failure	Heart failure (HF)
[106]	Evans et al., 2016	USA	Remote Health Monitoring for Older Adults and Those with Heart Failure: Adherence and System Usability	Heart failure (HF)

Refere nces	Author et al., Year	Country or Countries	Title(s)	Chronic disease(s)
[87]	Fritschi et al., 2022	USA	“Something Tells Me I Can’t Do That No More”: Experiences With Real-Time Glucose and Activity Monitoring Among Underserved Black Women With Type 2 Diabetes	Type 2 diabetes
[62]	Fukuoka et al., 2015	USA	A Novel Diabetes Prevention Intervention Using a Mobile App: A Randomized Controlled Trial With Overweight Adults at Risk	Type 2 diabetes (and overweight)
[41,42, 114]	Goldenthal et al., 2019<br>Caceres et al., 2020<br>Masterson Creber et al., 2022<br>(The iHEART Study)	USA	(2019) Recurrent atrial fibrillation/flutter detection after ablation or cardioversion using the AliveCor KardiaMobile device: iHEART results<br><br>(2020) Mobile Electrocardiogram Monitoring and Health-Related Quality of Life in Patients With Atrial Fibrillation Findings From the iPhone Helping Evaluate Atrial Fibrillation Rhythm Through Technology (iHEART) Study<br><br>(2022) Cardiac symptom burden and arrhythmia recurrence drives digital health use: results from the iHEART randomized controlled trial	Atrial fibrillation (AF) or atrial flutter (AFL)
[43,115]	Hickey et al., 2017<br>Reading et al., 2018	USA	Evaluating the Utility of Mhealth ECG Heart Monitoring for the Detection and Management of Atrial Fibrillation in Clinical Practice (2017)<br><br>Factors Influencing Sustained Engagement with ECG Self-Monitoring: Perspectives from Patients and Health Care Providers (2018)	Recurrent atrial fibrillation (AF) or other atrial arrhythmias
[107]	Ho et al., 2021	Canada	Testing the Feasibility of Sensor-Based Home Health Monitoring (TEC4Home) to Support the Convalescence of Patients With Heart Failure: Pre-Post Study	Heart failure (HF)
[63]	Ito et al., 2022	Japan	Daily self-monitoring of blood pressure decreases systolic and diastolic blood pressure in hypertensive participants	Hypertension
[64]	Janevic et al., 2020	USA	Acceptability and Effects of Commercially Available Activity Trackers for Chronic Pain Management Among Older African American Adults	Chronic musculoskeletal pain
[65]	Jiwani et al., 2022	USA	A Behavioral Lifestyle Intervention to Improve Frailty in Overweight or Obese Older Adults with Type 2 Diabetes: A Feasibility Study	Type 2 diabetes (and overweight/obese and ≥ 65 years)
[124]	Kayyali et al., 2016	4 participating clinical sites in Greece, the United Kingdom, Ireland and the Netherlands	Qualitative investigation into a wearable system for chronic obstructive pulmonary disease: the stakeholders' perspective	Chronic obstructive pulmonary disease (COPD) with comorbidities

References	Author et al., Year	Country or Countries	Title(s)	Chronic disease(s)
[127]	Khusial et al., 2020	The Netherlands and the United Kingdom	Effectiveness of myAirCoach: A mHealth Self-Management System in Asthma	Asthma
[88]	Kim et al., 2016	Republic of Korea	Feasibility of a Patient-Centered, Smartphone-Based, Diabetes Care System: A Pilot Study	Type 2 diabetes
[83]	Kim et al., 2019	Republic of Korea	The Effect of a Smartphone-Based, Patient-Centered Diabetes Care System in Patients With Type 2 Diabetes: A Randomized, Controlled Trial for 24 Weeks	Type 2 diabetes
[66]	Kooiman et al., 2018	The Netherlands	Self-tracking of Physical Activity in People With Type 2 Diabetes	Type 2 diabetes
[121]	Koole et al., 2019	The Netherlands	First real-world experience with mobile health telemonitoring in adult patients with congenital heart disease	Congenital heart disease
[67]	Krein et al., 2013	USA	Pedometer-Based Internet-Mediated Intervention For Adults With Chronic Low Back Pain: Randomized Controlled Trial	Chronic low back pain
[92]	Lakshminarayan et al., 2018	USA	A mHealth-based care model for improving hypertension control in stroke survivors: Pilot RCT	Hypertension (control in stroke survivors)
[68]	Lee et al., 2021	Republic of Korea	Therapeutic Exercise Platform for Type-2 Diabetic Mellitus	Type 2 diabetes
[108]	Lefler et al., 2018	USA	Evaluating the Use of Mobile Health Technology in Older Adults With Heart Failure: Mixed-Methods Study	Heart failure
[69]	Li et al., 2018	Canada	Efficacy of a Community-Based Technology-Enabled Physical Activity Counseling Program for People With Knee Osteoarthritis: Proof-of-Concept Study	Knee osteoarthritis
[117]	Li et al., 2020	Taiwan	Mobile Health App With Social Media to Support Self-Management for Patients With Chronic Kidney Disease: Prospective Randomized Controlled Study	Chronic Kidney Disease at stages 1-4
[70]	Li et al., 2020	Canada	Efficacy of a Physical Activity Counseling Program With Use of a Wearable Tracker in People With Inflammatory Arthritis: A Randomized Controlled Trial	Rheumatoid arthritis (RA) or systemic lupus erythematosus (SLE)
[71]	Li et al., 2021	China	Efficiency of an mHealth App and Chest-Wearable Remote Exercise Monitoring Intervention in Patients With Type 2 Diabetes: A Prospective, Multicenter Randomized Controlled Trial	Type 2 diabetes
[103]	Mallow et al., 2018	USA	The effectiveness of mI SMART: A nurse practitioner led technology intervention for multiple chronic conditions in primary care	Diagnosis of chronic conditions that could be monitored and treated using this technology (i.e. any combination of

References	Author et al., Year	Country or Countries	Title(s)	Chronic disease(s)
				diabetes, hypertension, depression, or hyperlipidemia)
[93]	Marvel et al., 2021	USA	Digital Health Intervention in Acute Myocardial Infarction	Acute myocardial infarction (AMI)
[128]	Mosnaim et al., 2021	USA	The Impact of Patient Self-Monitoring Via Electronic Medication Monitor and Mobile App Plus Remote Clinician Feedback on Adherence to Inhaled Corticosteroids: A Randomized Controlled Trial	Asthma
[72]	Moy et al., 2015	USA	An Internet-Mediated Pedometer-Based Program Improves Health-Related Quality-of-Life Domains and Daily Step Counts in COPD: A Randomized Controlled Trial	Diagnosis of COPD, emphysema or chronic bronchitis
[129]	Munster-Segev et al., 2017	Israel	Incorporation of a Stress Reducing Mobile App in the Care of Patients With Type 2 Diabetes: A Prospective Study	Type 2 diabetes
[122]	Noble et al., 2016	United Kingdom (Isle of Wight)	Medication adherence and activity patterns underlying uncontrolled hypertension: Assessment and recommendations by practicing pharmacists using digital health care	Hypertension
[100]	Oh et al., 2022	Republic of Korea	Effect of an Integrative Mobile Health Intervention in Patients With Hypertension and Diabetes: Crossover Study	Diabetes type 2, hypertension and obesity
[101]	Or et al., 2020	Hong Kong	Improving Self-Care in Patients With Coexisting Type 2 Diabetes and Hypertension by Technological Surrogate Nursing: Randomized Controlled Trial	Diabetes type 2 and hypertension
[73]	Orme et al., 2018	United Kingdom	Findings of the Chronic Obstructive Pulmonary Disease-Sitting and Exacerbations Trial (COPD-SEAT) in Reducing Sedentary Time Using Wearable and Mobile Technologies With Educational Support: Randomized Controlled Feasibility Trial	Chronic obstructive pulmonary disease (COPD)
[35,74]	Ostlind et al., 2022<br>Ostlind et al., 2021	Sweden	Promoting work ability with a wearable activity tracker in working age individuals with hip and/or knee osteoarthritis: a randomized controlled trial (2022/RCT)<br><br>Physical activity patterns, adherence to using a wearable activity tracker during a 12-week period and correlation between self-reported function and physical activity in working age individuals with hip and/or knee osteoarthritis (2021)	Hip and/or knee osteoarthritis
[109]	Park et al., 2019	USA	Impact on Readmission Reduction Among Heart Failure Patients Using Digital Health Monitoring: Feasibility and Adoptability Study	Heart failure (HF)
[45,75]	Park et al., 2021 (RCT)<br>Elnaggar et al., 2021 (Qualitative)	USA	RCT: Mobile health intervention promoting physical activity in adults post cardiac rehabilitation: pilot randomized controlled trial.<br><br>Qualitative: Applying Mobile Technology to Sustain Physical Activity After Completion of Cardiac Rehabilitation: Acceptability Study	Cardiac Rehabilitation (CR): After a major cardiac event, such as myocardial infarction or coronary revascularization.

References	Author et al., Year	Country or Countries	Title(s)	Chronic disease(s)
[76]	Paul et al., 2016	United Kingdom	Increasing physical activity in stroke survivors using STARFISH, an interactive mobile phone application: a pilot study	Stroke survivors
[77]	Rabbi et al., 2018	USA	Feasibility and Acceptability of Mobile Phone–Based Auto-Personalized Physical Activity Recommendations for Chronic Pain Self-Management: Pilot Study on Adults	Chronic back pain
[46,78]	Radhakrishnan et al., 2020<br>Radhakrishnan et al., 2021	USA	Usability Testing of a Sensor-Controlled Digital Game to Engage Older Adults with Heart Failure in Physical Activity and Weight Monitoring (2020)<br><br>Feasibility of a Sensor-Controlled Digital Game for Heart Failure Self-management: Randomized Controlled Trial (2021)	Heart failure (HF)
[110]	Rahimi et al., 2020	United Kingdom	Home monitoring with technology-supported management in chronic heart failure: a randomised trial	Heart failure (HF)
[95]	Read, 2014	Canada	Feasibility of the Diabetes and Technology for Increased Activity (DaTA) Study: A Pilot Intervention in High-Risk Rural Adults	Type 2 diabetes
[79]	Richardson et al., 2016	USA	A Comparative Effectiveness Trial of Three Walking Self-monitoring Strategies	At least one of the following conditions: diabetes, coronary artery disease (CAD), hypercholesterolemia, hypertension, or obesity (BMI >30).
[49,89]	Shaw et al., 2020 (*Observational study*)<br>Lewinski et al., 2021 (*Qualitative descriptive study*).	USA	*Observational study*:<br>Self-monitoring diabetes with multiple mobile health devices<br>*Qualitative descriptive study*:<br>Perceptions of Using Multiple Mobile Health Devices to Support Self-Management Among Adults With Type 2 Diabetes: A Qualitative Descriptive Study	Type 2 diabetes and "underserved as patients who are racial/ethnic minorities, low income, or Medicaid-eligible".
[99]	Sieverdes et al., 2013	USA	mHealth medication and blood pressure self-management program in Hispanic hypertensives: a proof of concept trial	Hypertension
[50,125]	Stamenova et al., 2020<br>van Lieshout et al., 2020	Canada	Technology-Enabled Self-Management of Chronic Obstructive Pulmonary Disease With or Without Asynchronous Remote Monitoring: Randomized Controlled Trial<br><br>Evaluating the Implementation of a Remote-Monitoring Program for Chronic Obstructive Pulmonary Disease: Qualitative Methods from a Service Design Perspective	Chronic obstructive pulmonary disease (COPD)
[84]	Tang et al., 2013	USA	Online disease management of diabetes: engaging and motivating patients online with enhanced resources-diabetes (EMPOWER-D), a randomized controlled trial	Type 2 diabetes

References	Author et al., Year	Country or Countries	Title(s)	Chronic disease(s)
[80]	Timurtas et al., 2022	Turkey	Technology-based and supervised exercise interventions for individuals with type 2 diabetes: Randomized controlled trial	Type 2 diabetes
[37,48, 85]	Torbjornsen et al., 2014 (a)<br>Holmen et al., 2014 (b)<br>Holmen et al., 2016 (c)<br>(RENEWING HEALTH Study)	Norway	a) A Low-Intensity Mobile Health Intervention With and Without Health Counseling for Persons With Type 2 Diabetes, Part 1: Baseline and Short-Term Results From a Randomized Controlled Trial in the Norwegian Part of RENEWING HEALTH<br><br>b) A Mobile Health Intervention for Self-Management and Lifestyle Change for Persons With Type 2 Diabetes, Part 2: One-Year Results From the Norwegian Randomized Controlled Trial RENEWING HEALTH<br><br>c) Stages of change for physical activity and dietary habits in persons with type 2 diabetes included in a mobile health intervention: the Norwegian study in RENEWING HEALTH	Type 2 diabetes
[39,40, 111]	Triantafyllidis et al., 2015 (a)<br>Rahimi et al., 2015 (b)<br>Chantler et al., 2016 (c)<br>(The SUPPORT-HF Study)	United Kingdom	a) A personalised mobile-based home monitoring system for heart failure: The SUPPORT-HF Study<br>b) A user-centred home monitoring and self-management system for patients with heart failure: a multi-centre cohort study<br>c) Creating connections - the development of a mobile-health monitoring system for heart failure: Qualitative findings from a usability cohort study	Heart failure (HF)
[36,47, 81]	Verwey et al., 2012<br>van der Weegen et al., 2015<br>Verwey et al., 2016	The Netherlands	Get moving: the practice nurse is watching you! A case study of the user-centred design process and testing of a web-based coaching system to stimulate the physical activity of chronically ill patients in primary care (2012)<br><br>It's LiFe! Mobile and Web-Based Monitoring and Feedback Tool Embedded in Primary Care Increases Physical Activity: A Cluster Randomized Controlled Trial (2015)<br><br>Process evaluation of physical activity counselling with and without the use of mobile technology: A mixed methods study (2016)	Chronic obstructive pulmonary disease (COPD) and type 2 diabetes (40–70-year-old patients)
[86]	Wang et al., 2018	USA	A Behavioral Lifestyle Intervention Enhanced With Multiple-Behavior Self-Monitoring Using Mobile and Connected Tools for Underserved Individuals With Type 2 Diabetes and Comorbid Overweight or Obesity: Pilot Comparative Effectiveness Trial	Type 2 diabetes (and comorbid overweight or obesity)
[130]	Wang et al., 2022	China	Adherence, Efficacy, and Safety of Wearable Technology-Assisted Combined Home-Based Exercise in Chinese Patients With Ankylosing Spondylitis: Randomized Pilot Controlled Clinical Trial	Ankylosing spondylitis

References	Author et al., Year	Country or Countries	Title(s)	Chronic disease(s)
[112]	Ware et al., 2020	Canada	Outcomes of a Heart Failure Telemonitoring Program Implemented as the Standard of Care in an Outpatient Heart Function Clinic: Pretest-Posttest Pragmatic Study	Heart failure (HF)
[96]	Welch et al., 2015	USA	Telehealth program for type 2 diabetes: usability, satisfaction, and clinical usefulness in an urban community health center	Type 2 diabetes
[51,126]	Whelan et al., 2021<br>Farmer et al., 2017	United Kingdom	Recruiting patients to a digital self-management study whilst in hospital for a chronic obstructive pulmonary disease exacerbation: A feasibility analysis (2021)<br>Self-Management Support Using a Digital Health System Compared With Usual Care for Chronic Obstructive Pulmonary Disease: Randomized Controlled Trial (2017)	Chronic obstructive pulmonary disease (COPD)
[113]	Zan et al., 2015	USA	Patient engagement with a mobile web-based telemonitoring system for heart failure self-management: a pilot study	Heart failure (HF)
[82]	Zaslavsky et al., 2019	USA	Use of a Wearable Technology and Motivational Interviews to Improve Sleep in Older Adults With Osteoarthritis and Sleep Disturbance: Pilot Study	Osteoarthritis
[94]	Zhang et al., 2020	China	Adherence with blood pressure monitoring wearable device among the elderly with hypertension: The case of rural China	Hypertension
[90]	Zheng et al., 2020	USA	Actual Use of Multiple Health Monitors Among Older Adults With Diabetes: Pilot Study	Type 2 diabetes
