# Supplementary material for: Wearable Devices for Supporting Chronic Disease Self-Management: Scoping Review
Source: Interact J Med Res. 2024 Dec 9;13:e55925. doi: 10.2196/55925 (PMC11667132; doi:10.2196/55925)
Supplement: Multimedia Appendix 3 [file ijmr_v13i1e55925_app3.pdf]

Characteristics of the studies with Mixed Methods Appraisal Tool scores.

References	Authors, Year	Study design	Chronic disease(s) population; intervention duration; IoT(s) devices	Outcomes/Effects	Quality assessment (MMAT)
[123]	Alharbey & Chatterjee, 2019	RCT<br><br>Qualitative research	Chronic obstructive pulmonary disease (COPD)<br>RCT: N=30<br>Qualitative interviews: n=4<br>1 month<br>Multicomponent system: Peripheral capillary oxygen saturation (SpO2) device.	Awareness level (+)<br>Self-efficacy (+)<br>Behavioral intention (+)	RCT: **<br>Qualitative: ***
[102]	Alnosayan et al., 2017	Mixed Methods	Heart Failure (HF)<br>N=8<br>6 months<br>Weighing scale, BP monitor and BGM	Usage patterns for weight, blood pressure, blood glucose, and symptoms reported by the patients (-)<br>Satisfaction with the system (QoL and the System Usability) (-)	***
[52]	Amorim et al., 2019	RCT	Chronic low back pain<br>N=68<br>6 months<br>WAT only	Self-reported walking (+) and physical activity goals (+)<br>Care-seeking (-)	****
[119]	Andersen et al., 2020	Qualitative	Chronic heart disease<br>N=27<br>Over a 49-week period<br>WAT, BP, ECG, sleep tracker (*Fitbit*) and weighing scale	Patients' experiences with technologies (-)	*****
[44,53]	Arbillaga-Etxarri et al., 2018 (RCT)<br>Koreny et al., 2019	RCT<br>Cohort study	Chronic obstructive pulmonary disease (COPD)<br>N=407<br>12 months<br>WAT	Between-group efficacy/physical activity levels (-)<br>Severe COPD exacerbations, functional exercise capacity, body composition, health-related quality of life, anxiety and depression(-)	RCT: ***<br>Cohort: ***
[54]	Arsand et al., 2015	Pre-post	Type 1 diabetes<br>N=6<br>2 weeks<br>WAT	Patients' experiences with the smartwatch application (-)	***
[116]	Athilingam et al., 2017	RCT	Heart failure (HF)<br>N=18<br>30 days<br>A chest strap/sensor worn that connects to the Android device via Bluetooth to monitor physiological data, including heart rate, heart rate variability, and accelerometer data	Mean difference in HF outcomes (post-pre-Scores) and baseline versus 30-day follow-up scores: Self-care management (+), Self-care confidence (+), HF knowledge (+)<br>QoL in both groups (-)<br>Medication adherence in both groups (-)	***

References	Authors, Year	Study design	Chronic disease(s) population; intervention duration; IoT(s) devices	Outcomes/Effects	Quality assessment (MMAT)
[55]	Bailey et al., 2020	Cohort study	Chronic knee (n=3796) and back pain (n=6468)<br>N = 10 264<br>12 weeks<br>WAT (wearable motion sensors with straps)	Pain (-)<br>Engagement levels, program completion, program satisfaction, condition-specific pain measures, depression, anxiety, and work productivity (-)	****
[56]	Baron et al., 2019	RCT	Prehypertension/Stage 1 Hypertension<br>N=16<br>6 weeks<br>Wearable sleep tracker	Sleep time/quality (+)<br>Blood pressure (+)<br>Decreased depressive symptoms (+)	**
[105]	Bennett et al., 2018	RCT	Hypertension, diabetes, hyperlipidemia and obesity<br>N=337<br>12 months<br>Weighing scale	Weight change (-)<br>≥ 5% weight loss, waist circumference, blood pressure, fasting lipids, glucose, and HbA1c changes (-)	****
[57]	Bentley et al., 2020	RCT (feasibilty)<br><br>Qualitative research	Chronic obstructive pulmonary disease (COPD)<br>N=30<br>Qualitative: n=16<br>8 weeks<br>WAT only	Physical activity (step count) (-)<br>Perceived self-efficacy (questionnaire-based) (-)	RCT: *<br>Qualitative: *****
[38,104]	Bloss et al., 2016<br>Kim et al., 2016	RCT<br>(and sub-analyses of RCT)	Hypertension, insulin-dependent or non-insulin dependent diabetes, and/or with arrhythmia<br>N=160<br>And a subset of n=95 with hypertension<br>6 months<br>BP, BG, and ECG monitoring	Main RCT: Health self-management (-), health care resource utilization (-)<br><br>Sub-analyses of RCT: Improvements in hypertensive patient activation were associated (+) with improvements in cigarette smoking and BP control	RCT and sub study: ****
[120]	Broers et al., 2020	RCT	Cardiac patients (heart failure, coronary artery disease, hypertension)<br>N=150<br>6 months<br>WAT, BP, ECG, sleep tracker (*Fitbit*), and weighing scale	Life-style behavior (+) and QoL over time (-)	*
[97]	Chandler et al., 2019	RCT	Hypertension<br>N=54<br>9 months<br>BP monitor and electronic medication tray/device	Blood pressure (+)<br>Medication adherence (-)	***
[58]	Chhabra et al., 2018	RCT	Chronic low back pain<br>N=93<br>12 weeks<br>WAT only	Pain (+) and disability (+)<br>Symptom score (+)<br>Physical activity (-)	****

References	Authors, Year	Study design	Chronic disease(s) population; intervention duration; IoT(s) devices	Outcomes/Effects	Quality assessment (MMAT)
[59]	Colomina et al., 2021	RCT	Complex chronic patients with osteoarthritis undergoing primary hip or knee arthroplasty<br>N=59<br>3 months<br>+follow-up at 6 months<br>WAT only	Health status: Between groups (-), intragroup (+);<br>Unplanned hospital visits and admissions during a 6-month follow-up (-)<br>The incremental cost-effectiveness ratio (-)	***
[91]	Culhane-Pera et al., 2022	Qualitative (formative study)	Hypertension<br>N=50<br>mHealth-based HTN care model explained by researchers to Hmong and Latino patients (n=45) and staff members (n=5) followed by open-ended interviews. BP monitor only	Hypertension knowledge/participants perspectives about a mHealth-based care model (-)	***
[118]	Dadosky et al., 2018	Prospective non-randomized trial	Heart failure (HF)<br>Case N=49<br>Control N=93 (historical control group)<br>30-day time period<br>A chest strap/sensor worn that connects to the Android device via Bluetooth to monitor physiological data, including heart rate, heart rate variability, and accelerometer data	Rehospitalization rates (-)<br>Patient self-care knowledge and technology satisfaction (-)	**
[98]	Davidson et al., 2015	RCT	Hypertension<br>N=43<br>6 months<br>BP monitor and electronic medication tray/device	Blood pressure control (+)	***
[34,60]	de Batlle et al., 2020<br>de Battle et al., 2021	Non-randomised experimental study<br><br>Economic evaluation	2 different use cases (UCs): a history of hospitalizations for chronic obstructive pulmonary disease or heart failure (use case [UC] 1),: n=48 or a scheduled major elective hip or knee arthroplasty (use case [UC] 2) n=29.<br>N=77<br>3 months (2020)<br>6 months (2021)<br>A set of integrated sensors (WAT)	Person-centeredness and continuity of care (2020) (-)<br>Acceptability and usability (2020) (-)<br>Changes in health status (2021): Intragroup (+), between-groups (-)<br>Unplanned hospital visits (+) and admissions during a 6-month follow up (2021) (-)<br>The incremental cost-effectiveness ratio (ICER) (2021) (-)	**
[61]	Deka et al., 2019	RCT	Heart failure (HF)<br>N=30<br>8 weeks<br>WAT (*Fitbit Charge HR*)	Objective physical activity feedback (IoT related outcome) on adherence to recommended exercise guideline (-)<br>Effects of group social support by internet-based synchronized face-to-face video (not IoT related outcome) (-)	****

References	Authors, Year	Study design	Chronic disease(s) population; intervention duration; IoT(s) devices	Outcomes/Effects	Quality assessment (MMAT)
[106]	Evans et al., 2016	Non-randomised experimental study	Heart failure (HF)<br>N=21<br>6 months<br>Weight scale and BP monitor	Usability/Adherence (-)	*
[87]	Fritschi et al., 2022	Qualitative	Type 2 diabetes<br>6 weeks<br>N=8<br>BGM + WAT	Self-regulation behaviors (-)	*****
[62]	Fukuoka et al., 2015	RCT	Type 2 diabetes (and overweight)<br>N=61<br>5 months<br>WAT only (waist)	Change in weight and BMI from baseline to 5-month follow-up (+)<br>Other clinical indicators: hip circumference (+), blood pressure (+), lipid profile (-), and glucose levels (-)	****
[41,42,114]	Goldenthal et al., 2019<br>Cacares et al., 2020<br>Masterson Creber et al., 2022<br>(The iHEART Study)	RCT	Atrial fibrillation (AF) or atrial flutter (AFL)<br>N=238<br>6 months<br>HR/ECG monitoring only	Detection of AF/AFL recurrence (2019) (+)<br>HRQoL in patients with AF (2020) (-)<br>Predictors of ECG monitor usage among patients with AF enrolled in the intervention (2022) (-)	**
[43,115]	Hickey et al., 2017<br>Reading et al., 2018	Non-randomised experimental study (a pilot cohort)<br><br>Qualitative research	Recurrent atrial fibrillation (AF) or other atrial arrhythmias<br>N=23<br>(and a control group of n=23 /age and gender matched).<br>Qualitative: n=21<br>6 months<br>HR/ECG monitoring only	Detection of recurrent AF or other atrial arrhythmias over a 6-month period of time (2017) (+)<br>QoL assessments (2017): PCS scores increased significantly) (+) while MCS scores (-) did not change significantly from baseline to 6 months.<br>Physical functioning, role physical, vitality, and mental health domain scores at 6 months (+)<br>Qualitative study: identification of barriers to sustained engagement or strategies to intervene upon engagement through application design (-)	Cohort study: **<br>Qualitative: *****
[107]	Ho et al., 2021	Pre-post/<br>Mixed methods	Heart failure (HF)<br>N=70<br>Qualitative interviews n=11<br>60 days post discharge<br>(+follow-up at 90 days)	Unscheduled ED revisits, readmission to hospital, and overall length of hospitalization (-)<br>Patient QoL, self-efficacy, end-user experience (-)<br>Health system cost-effectiveness including cost reduction and hospital bed capacity (-)	***

References	Authors, Year	Study design	Chronic disease(s) population; intervention duration; IoT(s) devices	Outcomes/Effects	Quality assessment (MMAT)
			Weighing scale and BP monitor	Qualitative: Contribution to a sense of safety and security after hospital discharged (-)<br>Involvement in self-care management (-)	
[63]	Ito et al., 2022	RCT	Hypertension (HTN)<br>N=161<br>2 months<br>Daily step count, stair-climbing, distance covered, HR and sleeping time were monitored with wearable devices/sensors (*Fitbit Charge 2*).	Between-groups Systolic BP decreased (+)<br>Daily self-monitoring decreased the BP of participants with HTN (-), but additional daily self-monitoring of body fat, sleeping time, and daily step count did not further decrease BP (-)	***
[64]	Janevic et al., 2020	Randomized controlled pilot and feasibility trial	Chronic musculoskeletal pain<br>N=51<br>Six weeks<br>WAT only	Between-groups improvement in functioning or walking (-)<br>Satisfaction with activity trackers (-), despite some technical or dexterity-related difficulties (-)	****
[65]	Jiwani et al., 2022	Cohort study<br><br>Feasibility study	Type 2 diabetes (and overweight/obese and ≥ 65 years)<br>N=20<br>6 months<br>WAT Only (wristband)	Feasibility: session attendance of a behavioral lifestyle Intervention (-)<br>Adherence to *Fitbit* usage to self-monitor diet and physical activity (-)<br>Preliminary efficacy of the intervention on frailty (+), physical function (-), QoL (-), and T2D-related outcomes (weight and BMI) (+)	***
[124]	Kayyali et al., 2016 The WELCOME system	Qualitative research	Chronic obstructive pulmonary disease (COPD) with comorbidities.<br>Patients with COPD: n=32<br>Informal carers: n=27<br>HCPs: n=23<br>Intervention duration prior interviews/focus groups: not mentioned/not found<br>Multicomponent system (wearable vest): a number of patient-held devices such a BG meter and inhaler monitoring device	Consensus reached on the duration of a wearable vest (ranging from daily to wearing whenever unwell (-)<br>*Most respondents disagreed with continuous wear, as it may be too stressful for them (-)	****
[127]	Khusial et al., 2020	2 cases:<br>RCT<br>Cohort/Pre-post	Asthma<br>RCT: N=30 (3-6 months)<br>Pre-post: N=12 (3 months).<br>an inhaler adapter, an indoor air-quality monitor, a WAT, a portable spirometer, a fraction exhaled nitric oxide device	Asthma control (+)<br>Exacerbations, QoL, and technology acceptance (+)	RCT: ***<br>Cohort/Pre-post: ***

References	Authors, Year	Study design	Chronic disease(s) population; intervention duration; IoT(s) devices	Outcomes/Effects	Quality assessment (MMAT)
[88]	Kim et al., 2016	Pre-post/a single-arm pilot study	Type 2 diabetes<br>N=30<br>12 weeks<br>BGM + WAT	Change in HbA1c at 12 weeks of intervention compared with baseline (+)<br>Summary of diabetes self-care activities (SDSCA) scores: including diet, exercise, and glucose monitoring components (+)<br>(* "*particularly in the upper tertile of HbA1c reduction*".	***
[83]	Kim et al., 2019	RCT	Type 2 diabetes<br>N=172<br>24 weeks<br>BGM	Between-groups difference of the change in HbA1c at 24-week (+)<br>Blood pressure (-), body weight (-), fasting plasma glucose (+), and lipid profile (-)<br>Between-groups event numbers of severe hyperglycemia and hypoglycemia (-)	**
[66]	Kooiman et al., 2018	RCT	Type 2 diabetes<br>N=72<br>12 weeks<br>WAT	Physical activity (average steps per day) (+)<br>Glycemic control, weight, body mass index [BMI], waist-hip ratio (-)<br>Participants satisfaction with technology (-)	****
[121]	Koole et al., 2019	Cohort study/an observational prospective registry	Congenital heart disease<br>Follow-up data available (N = 54)<br>Mean follow-up: 3 months<br>BP, ECG/Heart Rate (HR), and weighing scale	Recruitment: 55 (out of 129 CHD invited patients) participated (-)<br>Mean follow-up was 3.0 months (-)<br>Adherence was 97% (-)	**
[67]	Krein et al., 2013	RCT	Chronic low back pain<br>N=229<br>12 months<br>WAT only	Chronic back pain-related disability: at 6 months (+), at 12 months (-)	****
[92]	Lakshminarayan et al., 2018	RCT	Hypertension (control in stroke survivors)<br>N=50<br>6 months<br>BP monitor only	Hypertension control efficacy (-)	***
[68]	Lee et al., 2021	Non-randomised experimental study	Type 2 diabetes<br>N=10<br>At least 3 months<br>WAT	Effectiveness (use and satisfaction) of the proposed platform (-)	**
[108]	Lefler et al., 2018	Mixed methods	Heart failure<br>N=28 (Allocated in 3 groups)<br>Group 1: mHealth group (IoTs + connected to a 24-hour call center); n=7<br>Group 2: IoTs only group (not connected to a call center); n=11<br>Group 3; usual care n=10	Perspectives of older adults with HF:<br>Technological (IoT) groups daily monitoring post intervention was 100% (-)<br>Technology anxiety/fear changes post intervention (-)<br>Technology usability (ease of use) post intervention (-)	***

References	Authors, Year	Study design	Chronic disease(s) population; intervention duration; IoT(s) devices	Outcomes/Effects	Quality assessment (MMAT)
			12 weeks<br>Quantitative: 3 surveys<br>Qualitative: 19 interviews via telephone with 2 more performed in-person.<br>BP monitor and weighing scale	Participants communication and engagement with providers (-), but with many access problems (-)<br>Narrative/qualitative) data:<br>Traditional communication and engagement with providers prevailed, delaying access to care (-)<br>Technological (IoT) groups feeling secure knowing that someone was observing them (-)<br>Technological (IoT) groups feeling more confident in self-monitoring and managing (-)<br>Uncertainty and frustration with persistent health problems (-)	
[69]	Li et al., 2018	RCT	Knee osteoarthritis<br>N=61<br>4-6 months<br>*The delayed group received the same intervention than the IG 2 months later. Participants were assessed at baseline (T0) and at the end of 2 months (T1), 4 months (T2), and 6 months (T3)".<br>WAT (*Fitbit Flex*)	Mean time on moderate-to-vigorous physical activity (MVPA ≥3 metabolic equivalents [METs] (-)<br>Mean time on MVPA ≥4 METs, mean daily steps, mean time on sedentary activities, and Knee Injury and Osteoarthritis Outcome Score (KOOS) (-)	****
[117]	Li et al., 2020	RCT	Chronic Kidney Disease at stages 1-4<br>N=49<br>90 days<br>ECG, WAT (wristband), and sleep tracker	Participants self-efficacy and self-management (+)<br>QoL (-)<br>Number of steps (-)<br>The estimated glomerular filtration rate (eGFR) (+)<br>Body weight and percentage body fat (-)	****
[70]	Li et al., 2020	RCT	Rheumatoid arthritis (RA) or systemic lupus erythematosus (SLE)<br>N=118<br>27 weeks<br>WAT only	Pain (+) and perceived walking habit (+)<br>Step count, and self-management (-)<br>Post hoc analysis in participants with rheumatoid arthritis (RA):<br>MVPA (+) and pain (+)	****
[71]	Li et al., 2021	RCT	Type 2 diabetes<br>N= 101<br>3 months<br>WAT (chest worn)/ECG monitoring	Body fat percentage (+) and cardiorespiratory endurance (+)<br>Blood glucose level (-) insulin level (-). homeostasis model assessment of insulin resistance (HOMA-IR) (-), muscle strength (-), and cholesterol level (-)	***
[103]	Mallow et al., 2018	Pre-post	Diagnosis of chronic conditions that could be monitored and treated using the *mI SMART* technology intervention. For example, any combination of diabetes, obesity, hypertension, depression, or hyperlipidemia.<br>N=30	Blood glucose, blood pressure, and weight (-)	****

References	Authors, Year	Study design	Chronic disease(s) population; intervention duration; IoT(s) devices	Outcomes/Effects	Quality assessment (MMAT)
			12 weeks<br>BGM, BP and weighing scale		
[93]	Marvel et al., 2021	Nonrandomized controlled trial with a historical control	Acute myocardial infarction (AMI)<br>IG: n=200<br>CHG: n=864<br>30-days post-discharge<br>BP only	All-cause 30-day readmissions (-)<br>ED Visits (-)<br>HTN control efficacy (+)	****
[128]	Mosnaim et al., 2021	RCT	Asthma<br>N=100<br>12 weeks<br>a small electronic medication monitor (EMM) attached on the ICS and SABA asthma inhalers	Inhalers (ICS and SABA) use (+)	****
[72]	Moy et al., 2015	RCT	Diagnosis of COPD, emphysema or chronic bronchitis<br>N=239<br>4 months<br>WAT	HRQoL (-)<br>Daily step counts (+)	****
[129]	Munster-Segev et al., 2017	Single-arm pilot study	Type 2 diabetes<br>N=9<br>16 weeks<br>Photoplethysmography signal	Changes on weight (-), blood pressure (-), and glycemic measures of patients with T2D (-) Patients feedback/qualitative experience (-)	**
[122]	Noble et al., 2016	Non-randomised experimental study	Hypertension<br>N=39<br>2 weeks<br>"Devices that passively captured and shared information about medication-taking using an ingestible sensor, and daily patterns of rest, activity, and exercise using a wearable patch that incorporates an accelerometer".	Blood pressure (-)<br>Patient and provider experiences with a digital health feedback system (DHFS) (-)	*****
[100]	Oh et al., 2022	RCT<br>Crossover study	Diabetes type 2, hypertension and obesity<br>N=32<br>6 months<br>BGM and BP	The effects on body fat, blood pressure, and blood glucose levels (clinical outcomes) (-)<br><br>Satisfaction about the functions of the smartphone apps for the input of food intake, exercise, and taking medicine) (-)	**
[101]	Or et al., 2020	RCT	Diabetes type 2 and hypertension<br>N=299<br>24 weeks<br>BGM and BP	Between-groups changes in BG (HbA1c) and BP (SBP and DBP) (-)<br>Intragroup changes in BG (HbA1c) and BP (SBP and DBP) (+)	****

References	Authors, Year	Study design	Chronic disease(s) population; intervention duration; IoT(s) devices	Outcomes/Effects	Quality assessment (MMAT)
			* *"Control group participants were supplied with BG and BP monitors of the same type, with logbooks as specified under conventional self-management protocols"*.	Between-groups changes in medication adherence, general adherence to treatment, adherence to disease-specific activities, diabetes knowledge and hypertension knowledge (-)<br>Intragroup changes in medication adherence, general adherence to treatment, adherence to disease-specific activities, diabetes knowledge and hypertension knowledge (+)	
[73]	Orme et al., 2018	RCT	Chronic obstructive pulmonary disease (COPD)<br>N=33<br>2 weeks<br>WAT (waist-worn)	Feasibility outcomes: Eligibility, uptake, and retention (-)<br>Acceptability, behavioral responses to vibration prompts (-)	*
[35,74]	Ostlind et al., 2022<br>Ostlind et al., 2021	RCT	Hip and/or knee osteoarthritis<br>N=124<br>12 weeks<br>WAT (wrist-worn)	Baseline and 3-month follow-up differences: Between-groups differences for presenteeism and work productivity loss at 3-month follow-up (+)<br>Between-groups differences for presenteeism and work productivity loss at 6- and 12-month follow-up (-)	**
[109]	Park et al., 2019	Non-randomised experimental study	Heart failure (HF)<br>N=58<br>30-day post discharge.<br>Weighing scale and BP monitor	30-day all-cause readmission (+)<br>Usage of the monitors (-)	***
[45,75]	Park et al., 2021 (RCT)<br>Elnaggar et al., 2021 (Qualitative)<br><br>The Mobile4Heart study	RCT<br><br>Qualitative research	Cardiac Rehabilitation (CR): After a major cardiac event, such as myocardial infarction or coronary revascularization.<br>RCT N=60<br>2 months<br>Qualitative: n=7<br>WAT (*Fitbit and Movn mobile app)*	Mean daily step count difference between groups (+)<br>Between-groups differences for the 6MWT, depression, or self-efficacy to maintain exercise (-)<br>Levels of satisfaction with technologies (-)	RCT: ****<br>Qualitative: ****
[76]	Paul et al., 2016	RCT (pilot)	Stroke survivors<br>N=23<br>6 weeks<br>WAT	Average daily step count (+), walking time (+), fatigue (+)<br>Significant group–time interactions for other outcome measures (-)	**
[77]	Rabbi et al., 2018	Non-randomised experimental study	Chronic back pain<br>N=10	Use, acceptability, and early efficacy (-)<br>Acceptance toward forms of recommendations (-)	*****

References	Authors, Year	Study design	Chronic disease(s) population; intervention duration; IoT(s) devices	Outcomes/Effects	Quality assessment (MMAT)
			5 weeks<br>WAT only		
[46,78]	Radhakrishnan et al., 2020<br>Radhakrishnan et al., 2021	Mixed methods/Usability testing (2020)<br><br>RCT (2021)	Heart failure (HF)<br>N=10 (2020)<br>N=38 (2021)<br>1 week (2020)<br>12 weeks (2021)<br>And follow-up at 24 weeks (2021)<br>Sensor-Controlled Digital Game (SCDG), smart weighing scale, and a smartphone app (a narrative-driven game)	(2020) Overall Attitudes toward the SCDG (-)<br><br>Participants' engagement with the SCDG (-)<br>(2021) Feasibility : recruitment (-), retention (-), intervention engagement (-), and satisfaction (+)<br>Daily weight monitoring (-) and physical activity adherence (-)<br>HF knowledge and functional status (+), QoL (+), self-reported HF behaviors (+), motivation to engage in behaviors (+), and HF-related hospitalization (+)<br>The association between the number of days each player opened the game app with the number of days the player engaged in weight monitoring (+).<br>The association between the number of days each player opened the game app with the number of days with physical activity step data (+)<br><br>The association between the number of days each player opened the game app with the perceived usefulness (-)	Usability testing: N/A<br>RCT: ****
[110]	Rahimi et al., 2020	RCT	Heart failure (HF)<br>N=202<br>6 months<br>Weighing scale and BP monitor	The opportunity score defined by the use of guideline-recommended medical therapy for chronic HF and major comorbidities) (-)<br>Physical well-being of participants (-)	**
[95]	Read, 2014	Non-randomised experimental study	Type 2 diabetes:<br>N=25<br>8 weeks<br>BGM + WAT +BP monitor	Adherence rates for self-monitoring (-)<br>Stage of change (-), physical activity (-), and predicted VO2max (+)<br>Systolic and diastolic blood pressure (-), waist circumference (-), weight (-)<br>Participants experiences and perceptions of the technology (-)	***
[79]	Richardson et al., 2016	RCT	At least one of the following conditions: diabetes, coronary artery disease (CAD), hypercholesterolemia, hypertension, or obesity (BMI >30).<br>N=184	Change in weight at 6 months (kg) (+)<br>Change in HRQoL at 6 months (+)<br>Change in accelerometer-measured physical activity (-)	**

References	Authors, Year	Study design	Chronic disease(s) population; intervention duration; IoT(s) devices	Outcomes/Effects	Quality assessment (MMAT)
			6 months<br>WAT only		
[49,89]	Shaw et al., 2020<br>Lewinski et al., 2021	Observational study (2020)<br><br>Qualitative descriptive study (2021)	Type 2 diabetes and "underserved as patients who are racial/ethnic minorities, low income, or Medicaid-eligible".<br>N=60<br>6 months<br>Qualitative (n=20)<br>CGM + WAT +Weighing scale	Feasibility measures: assessment of total engagement in device submissions and survey completion over the 6 months of observation (-)<br>Participants' perception (qualitative) (-)	****
[99]	Sieverdes et al., 2013	RCT	Hypertension<br>N=10<br>3 months<br>BP monitor and electronic medication tray/device	Feasibility measures: 100% retention rates throughout the 3-month trial (-), medication adherence (-), on-time BP adherence values (-), resting (+) and ambulatory BP (-)	***
[50,125]	Stamenova et al., 2020<br>van Lieshout et al., 2020	RCT<br><br>Qualitative research	Chronic obstructive pulmonary disease (COPD)<br>N=122<br>6 months<br>Qualitative: 8 patients with COPD, 5 health care providers, and 3 hospital administrators). *"One remote-monitoring patient and 1 self-monitoring patient were interviewed together with their caregiver"*<br>Multicomponent system: "a Bluetooth-enabled device kit to monitor oxygen saturation, BP, temperature, weight, and symptoms".	Self-management skills, patient symptoms and health use: Improvement for all 3 groups at 6 months (including usual care) in Partners in Health (PIH) Scale (-)<br>Patients' symptoms measured with the St George's Respiratory Questionnaire (SGRQ) (-); and the Bristol COPD Knowledge Questionnaire (BCKQ) scores (-)<br>Between-groups differences on the SGRQ activity (-), symptom scores (-), hospitalizations (-), ED visits (-), or clinic visits (-)	RCT: ****<br>Qualitative: ****
[84]	Tang et al., 2013	RCT	Type 2 diabetes<br>N=415<br>12 months<br>BGM	Glucose and LDL cholesterol controls at 12 months (+)<br>BP, weight, or Framingham cardiovascular risk (-)<br>Medication management (+) ; Healthcare utilization (-)<br>Initiating online messages to providers, mostly Nurse Care Managers (+)<br>Knowledge about blood glucose testing (+)<br>Understanding of diabetes at 12 months (+)<br>Overall treatment satisfaction (+)<br>Willingness to recommend treatment to others at 12 months (+)	****
[80]	Timurtas et al., 2022	RCT	Type 2 diabetes<br>N=75<br>12 weeks<br>WAT	Change in glycemic control (HbA1c) (-)<br>Six Minute Walk Test, exercise behaviour, muscle function, and physical capacity (-)	***

References	Authors, Year	Study design	Chronic disease(s) population; intervention duration; IoT(s) devices	Outcomes/Effects	Quality assessment (MMAT)
[37 48,85]	Torbjornsen et al., 2014 (a)<br>Holmen et al., 2014 (b)<br>Holmen et al., 2016 (c)<br>(RENEWING HEALTH Study)	RCT	Type 2 diabetes<br>N=151<br>1 year<br>BGM	Glucose levels (HbA1c) at 4 months and after 1 year (-)<br>Between-groups differences of glucose levels (-)<br>Changes at 4 months in self-management measured using the health service navigation item in the *Health Education Impact Questionnaire, heiQ*) (+)<br>Mean change after 1 year of skills and technique acquisition after adjusting for age, gender, and education (-)<br>Other secondary outcomes: between groups differences in self-management, HRQoL, depressive symptoms, and lifestyle changes after 1 year (-)	****
[39,40,111]	Triantafyllidis et al., 2015 (a)<br>Rahimi et al., 2015 (b)<br>Chantler et al., 2016 (c)<br>(The SUPPORT-HF Study)	"Non-interventional cohort study"<br><br>Qualitative research	Heart failure (HF)<br>N=52<br>Qualitative (n=29)<br>"Median follow-up 6 months [inter-quartile range, IQR, 3.6–9.2])".<br>Weighing scale and BP monitor	Evaluation of system usage: (1) Patient adherence (the mean adherence in using the system was constantly high) (-)<br>(2) System effectiveness (the % of successfully completed self-monitoring sessions involving three steps as recorded within the usage logs) (-)<br>(3) System efficiency (the % of successfully completed sessions at the first attempt) (-)<br>Usability of the system (-)	Cohort: *****<br>Qualitative: *****
[36,47,81]	Verwey et al., 2012<br>van der Weegen et al., 2015<br>Verwey et al., 2016	RCT<br><br>Mixed Methods	Chronic obstructive pulmonary disease (COPD) and type 2 diabetes (40–70-year-old patients with)<br>RCT:<br>(Only patients)<br>N=199<br>4-6 month<br>Mixed Methods:<br>(Patients) N= 131<br>(Practice nurses) N=20<br>WAT only	RCT Primary outcome: The average minutes per day of physical activity per patient (van der Weegen et al., 2015) (+)<br>RCT Secondary outcomes: QoL/mental health score (+), and general self-efficacy/ exercise self-efficacy (-)<br>Mixed methods study outcome: the reach, implementation and satisfaction with the counselling protocol and the tool (-)	RCT: **<br>Mixed methods: ***
[86]	Wang et al., 2018	RCT	Type 2 diabetes (and comorbid overweight or obesity)<br>N=26<br>6 months<br>BGM	Difference on HbA1c at 6 months among the 3 groups (+)<br>Statistical group significance on percentage weight loss and HbA1c changes overtime (-)	**
[130]	Wang et al., 2022	RCT	Ankylosing spondylitis<br>N=54	Difference in the Ankylosing Spondylitis Disease Activity Score (ASDAS) (+)	****

References	Authors, Year	Study design	Chronic disease(s) population; intervention duration; IoT(s) devices	Outcomes/Effects	Quality assessment (MMAT)
			16 weeks<br>Photoplethysmography signal	Total pain, fatigue, spinal pain, and morning stiffness intensity (+)<br>Between-groups frequency of difficulty in high motivation at 16 weeks (+)<br>Between-groups differences for change in VO2 max, SF-36, back extensor endurance test, and the range of motion of cervical lateral flexion at 16 weeks (+)	
[112]	Ware et al., 2020	Pre-post	Heart failure (HF)<br>N=211<br>6 months<br>Weighing scale and BP monitor	Clinical outcomes (BNP values) (-)<br>QoL (+)<br>Patient self-care (+)<br>Health service utilization (+)	*
[96]	Welch et al., 2015	Pre-post	Type 2 diabetes<br>N=30<br>3 months<br>BGM + BP monitor + Electronic pillbox	Improvement in blood glucose control (+)<br>Usability, satisfaction and clinical usefulness (-)	***
[51,126]	Whelan et al., 2021<br>Farmer et al., 2017	RCT	Chronic obstructive pulmonary disease (COPD)<br>N=166<br>Until 6 months post-discharge<br>Multicomponent system: Self-monitoring devices, such as pulse oximeter (saturation of oxygen), and wrist-worn WAT.	RCT: Health status measured by the St. George's Respiratory Questionnaire specific questionnaire (SGRQ-C) at 12 months (-)<br>Relative risk of hospital admission for the IG (-)<br>Between-groups generic health status differences (+)<br>Between-groups median numbers of visits to practice nurses (+)<br>Between-groups median number of visits to general practitioners (-)<br>Feasibility study: Patient recruitment and rate of dropout (-)	***
[113]	Zan et al., 2015	Single-arm prospective pilot study	Heart failure (HF)<br>N=21<br>90 days<br>Weighing scale and BP monitor	Proximity and communication with health care team/doctors, and confidence about self-care management (-)<br>Heart failure-related QoLfrom baseline (-)<br>Overall engagement with the program (-). Hospital resource utilization (-)	***
[82]	Zaslavsky et al., 2019	Pre-post	Osteoarthritis<br>N=24<br>14 weeks (follow-up at 14 and 19 weeks)<br>WAT/Sleep tracker	Insomnia Severity Index (ISI) and Acceptance of Sleep Difficulties (ASD) (+)	****
[94]	Zhang et al., 2020	Non-randomised experimental study	Hypertension<br>N=212<br>1 month<br>BP monitor only	Adherence: measured with the “Compliance of Hypertensive Patients' Scale”, and the “Health-related Quality of Life Survey” (-)	***
[90]	Zheng et al., 2020	Non-randomised experimental	Type 2 diabetes<br>N=9	The actual use of technologies: PAT (-), blood glucose meter (-), and weighing scale (-)	***

References	Authors, Year	Study design	Chronic disease(s) population; intervention duration; IoT(s) devices	Outcomes/Effects	Quality assessment (MMAT)
		study/Single group study	3 months<br>CGM + WAT + Weighing scale	Changes in caloric intake from week 1 to 11 (-)<br>Changes in caloric intake at week 12 (-)<br>Mean daily step count from week 1 to 12 (-)<br>Mean % of weight loss (-)<br>Reduction of dose of oral hypoglycemic agents or insulin (-)	
Positive effect (identified as statistically significant) (+); Neutral (positive effect but not identified as statistically significant) (-); Neutral (-); Negative effect (-)					
