# Supplementary material for: Wearable Devices for Supporting Chronic Disease Self-Management: Scoping Review
Source: Interact J Med Res. 2024 Dec 9;13:e55925. doi: 10.2196/55925 (PMC11667132; doi:10.2196/55925)
Supplement: Multimedia Appendix 4 [file ijmr_v13i1e55925_app4.pdf]

Connected wearable devices used, outcomes measured, and effects per chronic disease cluster.

Diabetes (18 studies)

We identified eighteen studies in which participants with diabetes were recruited. Summary of the outcomes are presented in **Table 4**.

Table 4: Connected wearable devices used, outcomes measured, and effects

Outcomes / Connected wearable devices	Clinical	Behavioral	Patient technology experience	Holistic/ Biopsychosocial	Health system
Positive effect (identified as statistically significant) (+); Neutral (positive effect but not identified as statistically significant) (-); Neutral (-); Negative effect (-)					
Only blood glucose (BG) monitoring devices (but with more outcomes measured than only glycemic controls) [83,84,85,86]	Glucose level/HbA1c (+) [83,84,86]; (-) [85]<br><br>Cholesterol control (+) [84]<br><br>Body weight (-) [83]; (-) [84,86]<br><br>Blood pressure (-) [83,84]<br><br>Cardiovascular risk (-) [84]	Medication management (+) [84]<br>Knowledge about diabetes/BG testing (+) [84]<br>Skills and technique acquisition (-) [85]<br>Depressive symptoms and lifestyle changes (-) [85]	Overall treatment satisfaction (+) [84]<br>Willingness to recommend treatment to others (+) [84]	HRQoL (-) [85]	Initiating online messages to providers, mostly Nurse Care Managers (+) [84]<br>Healthcare utilization (-) [84]<br>Health service navigation self-management (+) [85]
BG + wearable activity trackers (WAT) [87,88]	Glucose level/HbA1c (+) [88]	Self-care activities scores (+) [88]<br>Self-regulation behaviors (-) [87]			
BG + WAT + Connected weighing scale (WS) [89,90]	Weight (-) [90]	Reduction of dose of oral hypoglycemic agents or insulin (-) [90]	Patient experience/satisfaction (-) [89]		

		Changes in caloric intake over time (-) [90]<br>Mean daily step count (-) [90]			
BG + WAT + Blood pressure (BP) devices [95]	VO2max (+) [95]<br>Weight (-) [95]<br>Waist/hip ratio/ hip circumference (-) [95]	Physical activity (-) [95]<br>Adherence rates for self-monitoring (-) [95]	Patient experience/satisfaction (-) [95]		
BG + Blood pressure (BP) + Electronic tray/pillbox devices [96]	Glucose level/HbA1c (+) [96]		Patient experience/satisfaction (-) [96]		
WAT devices only [54,62,65,66,68,71,80]	Glucose level/HbA1c (-) [62,66,71,80]<br>Frailty (+) [65]<br>Weight (+) [62,65]; (-) [66]<br>BMI (+) [62,65,71]; (-) [66]<br>Cardiorespiratory endurance (+) [71]<br>Waist/hip ratio/ hip circumference (+) [62]; (-) [66]<br>Blood pressure (+) [62]<br>Muscle strength/physical capacity (-) [71,80]<br>Cholesterol level (-) [71]	Physical activity (+) [66]<br><br>Session attendance of a behavioral lifestyle Intervention (-) [65]	Patient experience/satisfaction (-) [54,65,66,68]	QoL (-) [65]	
Photoplethysmography signal device [129]	Glucose level/HbA1c (-) [129]<br>Weight (-) [129] Blood pressure (-) [129]		Patient experience/satisfaction (-) [129]		

Heart failure (13 studies)

We identified thirteen studies in which participants with heart failure (HF) were recruited. Summary of the outcomes are presented in **Table 5**.

Table 5: Connected wearable devices used in heart failure studies, outcomes measured, and effects

Outcomes* / Connected wearable devices	Clinical	Behavioral	Patient technology experience	Holistic/ Biopsychosocial	Health system
Positive effect (identified as statistically significant) (+); Neutral (positive effect but not identified as statistically significant) (-); Neutral (-); Negative effect (-)					
Blood pressure (BP) and connected weighing scale (WS) devices [106-113]	BNP values (-) [112]	Self-efficacy/self-care management (+) [112]; (-) [107,108,113]<br>Daily monitoring post intervention (-) [108]<br>The use of guideline-recommended medical therapy (-) [110]	Usability and adherence to devices (-) [106,108,111]<br>End-user experience (-) [107]<br>Contribution to a sense of safety and security after hospital discharged (-) [107]<br>Usage of the devices (-) [109]	QoL (+)[112]; (-) [107,113]<br>Physical well-being of participants (-) [110]	Unscheduled ED revisits, readmission to hospital, and overall length of hospitalization (-) [107]<br>Health system cost-effectiveness including cost reduction and hospital bed capacity (-) [107]<br>Traditional communication and engagement with providers prevailed, delaying access to care (-) [108]<br>30-day all-cause readmission post discharge (+) [109]<br>Health service utilization (+) [112]<br>Proximity and communication with health care team/doctors (-) [113]<br>Hospital resource utilization (-) [113]
BP, WS, and blood glucose (BG) monitoring devices [102]	Usage patterns for weight, blood pressure, blood glucose, and symptoms reported by		Patient experience/satisfaction (-) [102]		

	the patients (-) [102]				
A chest strap [116] ECG + WAT (*accelerometer data*)/Small sensor worn on patient's chest wall [118] (ECG, BP, and WS)		Self-care management/confidence/ (+)[116]; (-) [118] Medication adherence (-) [116]	Patient experience/satisfaction (-) [118]	QoL (-) [116]	Rehospitalization rates (-) [118]
WAT only [61]		Adherence to recommended exercise guideline (-) [61]			
WAT and WS (with a digital game) [78]		HF knowledge and functional status (+)[78] Self-reported HF behaviors, motivation to engage in behaviors (+)[78]	Patient experience/satisfaction (-) [78]	QoL (+)[78]	HF-related hospitalization (+)[78]

Other cardiovascular conditions (10 studies)

We identified ten studies in which participants with cardiovascular diseases such as chronic heart disease (CHD), coronary artery disease (CAD), atrial fibrillation (AF) or atrial flutter (AFL), post-stroke control/cardiac rehabilitation, acute myocardial infarction (AMI), and any combination of heart failure and hypertension were recruited. Summary of the outcomes are presented in **Table 6**.

Table 6: Connected wearable devices used in studies, outcomes measured, and effects

Outcomes* / Connected wearable devices	Clinical	Behavioral	Patient technology experience	Holistic/ Biopsychosocial	Health system
Positive effect (identified as statistically significant) (+); Neutral (positive effect but not identified as statistically significant) (-); Neutral (-); Negative effect (-)					
Electrocardiogram (ECG) devices only [114,115]	Detection of atrial fibrillation (AF) or atrial flutter (AFL) recurrence (+) [114] Detection of recurrent AF or other atrial arrhythmias (+) [115]	Physical functioning, role physical, vitality, and mental health domain scores (+) [115]	HRQoL in patients with AF (-) [114]	Physical Component Summary (PCS) scores (+) [115] Mental Component Summary (MCS) scores (-) [115]	
WAT only [75,76]		Average/Mean daily step count/Walking time (+) [75,76] Fatigue (+) [76] 6MWT, depression, or self-efficacy to maintain exercise (-) [75]	Patient experience/satisfaction (-) [75]		

BP only<br>[92,93]		Self-efficacy (+) [93]; (-)<br>[92]			All-cause 30-day readmissions (-) [93]<br>ED Visits (-) [93]
BP, ECG, WS with [119,120]/or without WAT/Sleep tracker [121]		Life-style behavior (+)<br>[120]	Patient experience/satisfaction (-) [119]<br>Adherence (97%) to mHealth program (-)<br>[121]	QoL (-) [120]	
ECG, WAT, and sleep tracker [117]	The estimated glomerular filtration rate (eGFR) (+) [117]<br>Body weight/BMI (-)<br>[117]	Self-efficacy and self-management (+) [117]<br>Number of steps (-) [117]		QoL (-) [117]	

Hypertension (8 studies)

We identified eight studies in which participants with hypertension were recruited. Summary of the outcomes are presented in **Table 7**.

Table 7: Connected wearable devices used in studies, outcomes measured, and effects

**Outcomes*** / **Connected wearable devices**	**Clinical**	**Behavioral**	**Patient technology experience**	**Holistic/ Biopsychosocial**	**Health system**
Positive effect (identified as statistically significant) (+); Neutral (positive effect but not identified as statistically significant) (-); Neutral (-); Negative effect (-)					
Blood pressure (BP) and medication pillbox/tray devices [97,98,99]	Blood pressure/BP control (+) [97,99] Resting (+) and ambulatory BP (-) [99]	Medication adherence (-) [97,99]			
BP only [91,94]		Hypertension knowledge/participants perspectives about a mHealth-based care model (-) [91]	Factors associated with adherence to BP monitor (-) [94]		
WAT/Sleep tracker [56,63]	Blood pressure/Between groups Systolic BP (+) [56,63] Sleep time/quality (+) [56]	Decreased depressive symptoms (+) [56]			
An ingestible sensor, and a wearable patch that incorporates an accelerometer [122]	Blood pressure (-) [122]		Experiences with a digital health feedback system/ingestible sensor (-) [122]		Patients' experiences with pharmacists (-) [122]

Multimorbidity and other combination of chronic conditions (8 studies)

We identified eight studies that recruited participants with two or more chronic conditions. Summary of the outcomes are presented in **Table 8**.

Table 8: Connected wearable devices used in studies, outcomes measured, and effects

**Outcomes*** / **Connected wearable devices**	**Clinical**	**Behavioral**	**Patient technology experience**	**Holistic/ Biopsychosocial**	**Health system**
Positive effect (identified as statistically significant) (+); Neutral (positive effect but not identified as statistically significant) (-); Neutral (-); Negative effect (-)					
WAT only [60,79,81]	Change in weight (+) [79]	Minutes of physical activity daily (+) [81] Person-centeredness (-) [60] Change in accelerometer-measured physical activity (-) [79] General self-efficacy/ exercise self-efficacy (-) [81]	Acceptability and usability (-) [60] Patient experience/satisfaction (-) [81]	Intragroup changes in Health status (+)/ Between groups (-) [60] Change in HRQoL (+) [79] QoL/mental health score (+) [81]	Unplanned hospital visits (+) [60] Hospital admissions during follow-up (-) [60] Continuity of care (-) [60] The incremental cost-effectiveness ratio (-) [60]
BG and BP [100,101]	Intragroup changes (*both groups had wearable devices*) in BG (HbA1c) and BP (SBP and DBP) (+) [101]<br><br>Effects on body fat, blood pressure, and blood glucose levels (-) [100]	Intragroup changes (*both groups had wearable devices*) in medication adherence, general adherence to treatment, adherence to disease-specific activities, diabetes knowledge and hypertension knowledge (+) [101]	Patient experience/satisfaction (-) [100]		

BG, BP, and ECG [104]<br>BG, BP, and WS [103]	Blood glucose, blood pressure, and weight (-) [103]	Health self-management (-) [104]<br>*Improvements in hypertensive patient activation were associated (-) with improvements in cigarette smoking and BP control* [104]			Health care resource utilization (-) [104]
Connected weighing scale (WS) only [105]	Weight change (-)<br>≥ 5% weight loss, waist circumference, blood pressure, fasting lipids, glucose, and HbA1c changes (-) [105]				

Chronic obstructive pulmonary disease (8 studies)

We identified 8 chronic obstructive pulmonary disease (COPD) studies and summary of the outcomes are presented in **Table 9**.

Table 9: Connected wearable devices used in studies, outcomes measured, and effects

Outcomes* / Connected wearable devices	Clinical	Behavioral	Patient technology experience	Holistic/ Biopsychosocial	Health system
Positive effect (identified as statistically significant) (+); Neutral (positive effect but not identified as statistically significant) (-); Neutral (-); Negative effect (-)					
Multicomponent systems (monitoring of oxygen saturation) [123-126]<br>[123] SpO2 device only<br>[124] Inhaler adherence monitoring device + BG<br>[125] SpO2 + BP + WS<br>[126] SpO2 + WAT	Symptom scores (-) [125]	Awareness level (+) [123]<br>Self-efficacy (+) [123]<br>Behavioral intention (+) [123]<br>Self-management skills (-) [125]	Disagreement about continuous wear of a vest (-) **as it may be too stressful* [124]	Between groups generic health status differences (+) [126]	Median numbers of visits to practice nurses (+)/to general practitioners (-) [126]<br><br>Hospitalizations (-), ED visits (-), or clinic visits (-) [125,126]
WAT only [53,57,72,73]	Severe COPD exacerbations (-) [53]	Daily step count (+) [72]<br>Physical activity levels (-) [53]; Step count (-) [57]<br>Functional exercise capacity (-) [53]<br>Self-efficacy (-) [57]	Acceptability, responses to (waist-worn) vibration prompts (-) [73]	HRQoL (-) [53,72]<br>Anxiety and depression (-) [53]	

Chronic pain (6 studies)

We identified six studies on chronic pain and summary of the outcomes are presented in **Table 10**.

Table 10: Connected wearable devices used in studies, outcomes measured, and effects

Outcomes* / Connected wearable devices	Clinical	Behavioral	Patient technology experience	Holistic/ Biopsychosocial	Health system
Positive effect (identified as statistically significant) (+); Neutral (positive effect but not identified as statistically significant) (-); Neutral (-); Negative effect (-)					
Wearable activity trackers (WAT) only [52,55,58,64,67,77]	Symptom score (+) [58]<br><br>Disability (+) [58]<br><br>Pain (+) [58].<br>Pain (-) [55]<br><br>Chronic back pain-related disability at - 6 months (+) [67], at -12 months (-) [67]	Self-reported walking and physical activity goals (+) [52]<br>Engagement levels (-) [55]<br>Work productivity (-) [55]<br>Physical activity (-) [58]<br>Care-seeking/Self-management (-) [52]<br>Between-groups improvement in functioning/walking (-) [64]	Satisfaction of wearable motion sensors with straps (-) [55]<br>Use, acceptability, and early efficacy (-) [77]<br>Acceptance toward forms of recommendations (-) [77]<br>Satisfaction with activity trackers (-) [55,64] despite some technical or dexterity-related difficulties (-) [64]	Depression and anxiety (-) [55]	Care-seeking/ Consultations (-) [52]

Musculoskeletal conditions (6 studies)

We identified six studies on osteoporosis, rheumatoid arthritis, systemic lupus erythematosus or ankylosing spondylitis. Summary of the outcomes are presented in **Table 11**.

Table 11: Connected wearable devices used in studies, outcomes measured, and effects

**Outcomes*** / **Connected wearable devices**	**Clinical**	**Behavioral**	**Patient technology experience**	**Holistic/ Biopsychosocial**	**Health system**
Positive effect (identified as statistically significant) (+); Neutral (positive effect but not identified as statistically significant) (-); Neutral (-); Negative effect (-)					
WAT with [82]/or without sleep tracker [59,69,70,74]	Insomnia Severity Index (ISI) and Acceptance of Sleep Difficulties (ASD) (+) [82]<br><br>Pain (+) [70]<br><br>Knee Injury and Osteoarthritis Outcome Score/pain and symptoms (KOOS) (-) [69]	Perceived walking habits (+) [70]<br>Step count, and self-management (-) [70]<br>MVPA for participants with rheumatoid arthritis (+) [70]<br>Mean time on moderate-to-vigorous physical activity (MVPA ≥3 metabolic equivalents [METs] (-) [69]<br>Mean time on MVPA ≥4 METs, mean daily steps, mean time on sedentary activities (-) [69]<br>Knee Injury and Osteoarthritis Outcome Score/activities, sport, and recreation function (KOOS) (-) [69]		Health status: Intragroup (+); between groups (-) [59]<br>Knee Injury and Osteoarthritis Outcome Score/HRQoL (KOOS) (-) [69]	Unplanned hospital visits and admissions during a 6-month follow-up (-) [59]<br>The incremental cost-effectiveness ratio (-) [59]

		Differences for presenteeism and work productivity, loss at 3-month follow-up (+); at 6- and 12-month (-) [74]			
Photoplethysmography signal [130]	Difference in the Ankylosing Spondylitis Disease Activity Score (ASDAS) (+) [130]<br><br>Total pain, fatigue, spinal pain, and morning stiffness intensity (+) [130]<br><br>Dfferences for change in VO2 max, SF-36, back extensor endurance test, and the range of motion of cervical lateral flexion at 16 weeks (+) [130]	Between-groups frequency of difficulty in high motivation (+) [130]			

Asthma (2 studies)

We identified two studies on asthma and summary of the outcomes are presented in **Table 12**.

Table 12: Connected wearable devices used in studies, outcomes measured, and effects

Outcomes* / Connected wearable devices	Clinical	Behavioral	Patient technology experience	Holistic/ Biopsychosocial	Health system
Positive effect (identified as statistically significant) (+); Neutral (positive effect but not identified as statistically significant) (-); Neutral (-); Negative effect (-)					
Multicomponent system: an inhaler adapter, an indoor air-quality monitor, a WAT, a portable spirometer, and a fraction exhaled nitric oxide device [127]	Asthma control (+) [127] Exacerbations (+) [127]		Technology acceptance (+) [127]	QoL (+) [127]	
A small electronic medication monitor (EMM) attached on the ICS and SABA asthma inhalers [128]		Inhalers (ICS and SABA) use (+) [128]			
